# The relationship between metamotivational knowledge and performance

**DOI:** 10.3389/fpsyg.2023.1124171

**Published:** 2023-06-09

**Authors:** Jessica Ross, Tina Nguyen, Kentaro Fujita, David B. Miele, Michael C. Edwards, Abigail A. Scholer

**Affiliations:** ^1^University of Waterloo, Waterloo, ON, Canada; ^2^The Ohio State University, Columbus, OH, United States; ^3^Boston College, Chestnut Hill, MA, United States; ^4^Arizona State University, Tempe, AZ, United States

**Keywords:** metamotivation, motivation, self-regulation, regulatory focus, performance

## Abstract

Self-regulation research highlights the performance trade-offs of different motivational states. For instance, within the context of regulatory focus theory, promotion motivation enhances performance on eager tasks and prevention motivation enhances performance on vigilant tasks (i.e., regulatory focus task-motivation fit). Work on metamotivation—people’s understanding and regulation of their motivational states—reveals that, on average, people demonstrate knowledge of how to create such task-motivation fit; at the same time, there is substantial variability in this normative accuracy. The present research examines whether having accurate normative metamotivational knowledge predicts performance. Results revealed that more accurate metamotivational knowledge predicts better performance on brief, single-shot tasks (Study 1) and in a consequential setting (course grades; Study 2). The effect was more robust in Study 2; potential implications of this variability are discussed for understanding when and why knowledge may be associated with performance.

## Introduction

There is no way around it: succeeding at our goals can be difficult. Different goals place distinct performance demands on us (e.g., Freitas and Higgins, 2002), and they never stop calling. Yet responding effectively pays off: Individuals who navigate their task goals effectively experience benefits in a number of diverse areas, including higher life satisfaction, better interpersonal relations, and fewer health issues (Tangney et al., 2004). Because self-regulatory success plays such a crucial role in so many significant life outcomes, it is not surprising that there has been great interest in understanding what makes some people perform better on goal-related tasks than others.

Research has revealed a number of answers to when and why some people are more likely than others to succeed at their goals. Some approaches have focused on differences in general capacities or vulnerabilities, such as people’s general ability to regulate their thoughts, emotions, and behavior (i.e., trait self-regulation; Vohs and Baumeister, 2004), superior executive functions (Hofmann et al., 2012), or cue-reactivity (Boswell and Kober, 2016). Other approaches have looked outside the individual to general contextual factors that influence performance, such as the availability of temptations in one’s environment (Milyavskaya and Inzlicht, 2017) or social contexts that provide goal support (Briskin et al., 2019). Yet other approaches have focused on goal-related factors that improve performance, such as higher goal commitment (Locke et al., 1988), goal specificity (Locke and Latham, 1990), or the extent to which goals align with an individual’s interests and values (Sheldon and Elliot, 1999).

Beyond these factors, a nascent area of research is beginning to examine the contribution of meta-level motivational processes (i.e., metamotivation) to individuals’ self-regulatory -success and failure (Miele and Scholer, 2018; Scholer et al., 2018; Miele et al., 2020). This approach builds on work about individuals’ lay beliefs about the way the world works (e.g., Dweck, 2006) to suggest that people, based on their beliefs and knowledge about how motivation works, may take an active role in directing their motivation in ways that can support or undermine goal success. Thus, a metamotivational approach goes beyond viewing people as being either “good” or “bad” at self-regulating, and instead considers the types of tasks or situations that an individual might struggle with depending on their awareness of performance trade-offs, their repertoire of strategies, and their knowledge of how to identify and regulate these motivational states in themselves. Prior work has examined the nature of people’s motivational knowledge, but the current paper examines whether one specific form of this knowledge—people’s metamotivational beliefs about task-motivation fit in the domain of regulatory focus—predicts task performance.

### Metamotivational beliefs about regulatory focus task-motivation fit

Building on insights from the metacognition (Flavell, 1979; Nelson and Narens, 1990) and educational psychology literatures on motivation regulation (Schunk and Zimmerman, 1994; Wolters et al., 2011), the growing area of research on metamotivation examines what people know about managing both the quantity and quality of their motivation (Scholer et al., 2018). Metamotivation consists of two reciprocal processes—metamotivational monitoring, which involves assessing the motivation needed to pursue a goal successfully—and metamotivational control, which involves identifying and implementing strategies to upregulate or sustain desired motivational states (Miele and Scholer, 2018). Critically, the effectiveness of both monitoring and control is posited to rely on people’s beliefs about how motivation works. It is important to note that metamotivation research works under the assumption that this knowledge can operate tacitly or implicitly (Wagner and Sternberg, 1985), such that participants can demonstrate knowledge via their responses to the different scenarios even if they are not aware that they possess this knowledge or are unable to articulate it more explicitly.

Initial explorations of metamotivational knowledge examined what people believe about how different types of motivation fit tasks that vary in motivational affordances. There is a long tradition in motivation science of identifying qualitatively distinct types of motivation (Higgins, 1997; Deci and Ryan, 2000; Trope and Liberman, 2000). Evidence suggests that a given type of motivation can be normatively helpful, harmful, or irrelevant, depending on the situation (Higgins, 2000, 2006; Sansone, 2009; Fujita et al., 2019). For instance, research on regulatory focus theory (Higgins, 1997) has shown that performance on tasks that afford eager motivation (hereby referred to as eager tasks), which rely primarily on divergent or associative thinking (e.g., a brainstorming task) or a focus on the bigger picture (Förster and Higgins, 2005), tends to benefit from promotion motivation (a motivational state characterized by enthusiastically seeking opportunities for gains or growth; Friedman and Förster, 2001; though see Baas et al., 2011). In contrast, performance on tasks that afford vigilant motivation (hereby referred to as vigilant tasks) that require convergent thinking and attending to errors (e.g., proofreading a text) or carefully attending to concrete details (e.g., quality control inspections; Semin et al., 2005) tends to be enhanced by prevention motivation (a motivational state characterized by carefully protecting against potential losses or negative outcomes; Förster et al., 2003). In other words, there are times when either a promotion or prevention motivational state will lead to more optimal performance on a certain type of task, identified as regulatory focus *task-motivation fit* (Scholer and Miele, 2016).^1^

Scholer and Miele (2016) assessed what people knew about creating regulatory focus task-motivation fit by using a paradigm modeled after one used in research on instrumental emotion regulation (Ford and Tamir, 2012). Participants were presented with tasks that varied in their motivational affordances and were asked to report their preferences or performance expectancies for various preparatory activity-task combinations. Responses on this measure reveal participants’ metamotivational beliefs about different strategy-task combinations and can also be used to calculate the extent to which participant beliefs align with theory and prior empirical research and can, thus, be considered normatively accurate. Several studies using this assessment reveal that people, on average, do demonstrate normatively accurate knowledge of task-motivation fit in this domain, such that they endorse promotion-inducing recall activities for eager vs. vigilant tasks and prevention-inducing recall activities for vigilant vs. eager tasks (Scholer and Miele, 2016).

Although recent work suggests that there is some cross-cultural similarity in the normative accuracy of this knowledge (Nguyen et al., 2019), research also indicates that there is significant individual-level variability in normative accuracy, and that this variability can affect downstream outcomes. For instance, Scholer and Miele (2016) found that individuals’ metamotivational knowledge influenced their task selection decisions based on a given motivational state. Furthermore, Jansen et al. (2022) found that managers who demonstrated an awareness of regulatory focus task-motivation fit by motivating employees in ways consistent with task-motivation fit (e.g., using eager messages to motivate employees for a creativity task) were perceived as more effective leaders. In sum, prior work suggests that, on average, people have normatively accurate knowledge of regulatory focus task-motivation fit, and that this knowledge is associated with consequential behaviors. However, no work to date has examined whether normatively accurate metamotivational knowledge is related to better goal-relevant task performance, a critical self-regulatory question.

### The present research

The present research seeks to examine whether the normative accuracy of people’s metamotivational knowledge of regulatory focus task-motivation fit is associated with better performance. Studies 1a and 1b assessed participants’ metamotivational knowledge of task-motivation fit and examined whether normatively accurate knowledge of task-motivation fit was related to performance on brief, single-shot brainstorming and proofreading tasks (i.e., performance on tasks that are completed and assessed in a single, non-cumulative manner). Studies 2a and 2b extended this work by testing this relationship in a more naturalistic and consequential setting that unfolds over time—specifically, looking at participants’ final grades in an undergraduate psychology course. Examining performance in these distinct contexts allowed us to examine whether metamotivational knowledge is associated with self-regulatory success and whether there is variation in the strength of the relationship between knowledge and performance between these performance contexts (single-shot, low stakes laboratory performance vs. multi-shot, consequential performance). Materials for all studies and data for Study 1 are available on Open Science Framework.^2^ Study 2 contains students’ FERBA protected academic records, so all deidentified data are available upon request, by IRB approval.

## Study 1

Studies 1a and 1b were designed to test whether metamotivational knowledge of regulatory focus task-motivation fit predicts performance in single-shot lab tasks. First, in an initial session, participants completed the knowledge assessment measure created by Scholer and Miele (2016), in which they reported their preferences for engaging in different recall activities as preparatory exercises for eager and vigilant tasks. Then, in a second session, participants were randomly assigned to complete either an eager (brainstorming; Friedman and Förster, 2001) or vigilant (proofreading; Förster et al., 2003) task. We hypothesized that metamotivational knowledge of regulatory focus task-motivation fit would predict better task performance in the second session.

Given that Study 1b represents a near-direct replication of Study 1a, we present combined analyses for these studies (as we also do for Studies 2a and 2b). Combining the studies allows for more precise estimates of effect sizes and is consistent with recent recommendations to evaluate evidence across all data available to test hypotheses rather than individual studies (Fabrigar and Wegener, 2016). As we discuss in depth below, the observed effect differs for Study 1a versus 1b (to preview, there is no difference in the observed effects for Study 2a versus 2b); the detailed analyses for each sample are presented in the Supplementary material and we discuss potential interpretations in the study discussion.

### Materials and methods

#### Participants

Undergraduate participants at a large Canadian university (*N =* 336: Study 1a, *n* = 169, Study 1b, *n* = 167; *M*_age_ = 20.15, *SD*_age_ = 4.24. 245 women, 89 men, 4 did not report gender; 39.1% White/Caucasian, 22.8% East Asian, 20.1% South Asian, 4.4% Black/African, 4.1% Middle Eastern, 8.9% Bi-racial, Multi-racial, or other) completed a two-part online study in exchange for course credit. There were no significant main effects or interactions with gender, so this variable is not discussed further. Our goal was to recruit as many participants as possible over the course of each semester, especially given the possibility of attrition in this two-part study.^3^ With our final combined *N* of 336, we had 90% power to detect an effect as small as *f*^2^ = 0.03 for the primary analysis of our hypothesis—a linear multiple regression analysis (two-tailed, 8 predictors). The present study was part of a broader investigation; measures assessed in Session 1 unrelated to the current research questions are described in the Supplementary material. This study was reviewed and approved by the University of Waterloo Research Ethics Board, and participants provided their written informed consent to participate in both sessions of the study.

#### Procedure

In Session 1, participants completed a measure of regulatory focus metamotivational knowledge (Scholer and Miele, 2016). In Session 2, participants were randomly assigned to complete either a brainstorming or proofreading task. There were some variations in the length of time between sessions in the two samples. In Study 1a, the time between study sessions ranged from a few minutes to several weeks due to some idiosyncrasies related to the implementation of two-part studies in the participant pool. This issue was resolved in Study 1b, such that participants received a link to complete Session 2 three days after completing Session 1 and were told they had 7 days to complete it (Study1a: *M* = 12.65 days, *SD* = 17.87; Study 1b: *M* = 6.28 days, *SD* = 5.20). This was the only methodological difference between Studies 1a and 1b. Importantly, time between sessions did not affect the results (see Supplementary material). As also detailed in the Supplementary material, there was no difference in knowledge between those who completed both sessions versus Session 1 only, and no relation between knowledge and time between sessions. After completing the task, participants responded to task-related questions, were debriefed, and received course credit.

#### Materials

**Metamotivational Knowledge Assessment**. Participants completed an assessment of metamotivational knowledge of regulatory focus used in prior work (Scholer and Miele, 2016). Participants were told that they would see descriptions of tasks paired with a recall activity. For each pair, participants rated how much they would prefer to complete that recall activity (e.g., Please write about a time in the past when you felt you made progress toward being successful in life) before doing the task (e.g., Your goal is to imagine a future no one has seen before by seeing possibilities and occasions for advancement) on a scale from 1 (*not at all*) to 7 (*very much*). The regulatory focus knowledge assessment consisted of four tasks (2 eager, 2 vigilant) and 12 recall activities (4 promotion focus, 4 prevention focus, 4 neutral). Thus, participants saw a total of 48 randomly presented task and recall activity pairs. As described in Scholer and Miele (2016), vigilant and eager task descriptions and recall activities were constructed based on past empirical work (i.e., past work has shown that these activities induce promotion and prevention motivations; Freitas and Higgins, 2002). The complete text for the metamotivational knowledge assessment is available in the Supplementary material and by using the OSF link.

The Supplementary material also includes a psychometric assessment of the measure. In sum, a four-factor model, where each factor corresponded to a combination of task type and recall type, generally resulted in acceptable to good fit across samples. We also tested more complex models that attempted to capture the way in which the items corresponding to these factors were combined to form the single index of metamotivational knowledge used in our primary analyses (see below). With all of these models, there were issues with convergence and/or instability within or across samples. However, because there are potential reasons why such models may not have fit the data (e.g., knowledge may be better represented by formative measurement models; Stadler et al., 2021), we retained the analytic approach used in past work to examine individual differences in metamotivational knowledge (e.g., Scholer and Miele, 2016). The index associated with this approach exhibited good one-year test–retest reliability and strong discriminant validity (Study S1). For more detail about our psychometric assessment of the measure, see the Supplementary material.

**Task Performance.** In the second session of the study, participants were told: “On the next page you will be presented with a computer task designed to measure your performance. You will have 3 min to complete the task.” They were then randomly assigned as part of a between-participants manipulation to complete one of two of the following tasks:

***Eager Task (Brainstorming).*** The unusual uses task (Guilford, 1967; Friedman and Förster, 2001) asks participants to come up with as many creative ways to use an inanimate object as possible in 3 minutes. Participants were given the following instructions: “For the brainstorming task, list as many creative ways to use a TIN CAN as possible. The ideas you write down should be neither typical nor virtually impossible.” Performance was assessed using two metrics: number of ideas and originality ratings (Baas et al., 2011). The number of ideas was measured by the counting the total number of non-redundant ideas generated by each participant. These ideas were then individually coded for originality. Six trained coders (three per study) who were blind to the hypothesis evaluated each use independently and in random order on originality, on a scale from 1 (*not at all creative*) to 7 (*extremely creative*). Participant originality scores were created by averaging the ratings for each use they generated. Interrater reliability was good (Cicchetti, 1994), with an intra-class correlation coefficient of 0.68 for Study 1a and 0.80 for Study 1b. The two performance metrics were significantly but modestly correlated, *r*(161) = 0.23, *p* = 0.003. We created a composite creativity score (the average of the two scores) that we use in the primary analysis predicting overall performance. However, for full transparency—given the modest correlation and given that the results differ depending on the metric—we also present the results for each metric separately (number of ideas and originality).

***Vigilant Task (Proofreading)*.** The proofreading task involved a 400-word text discussing psychological theories of attraction (see Förster et al., 2003). The text contained a total of 46 errors and participants had 3 minutes to identify as many as possible. Participants were given the following instructions: “Please proofread the following text AS QUICKLY AND AS ACCURATELY as you can. Click on any word that contains an error (and no other words).” Performance was assessed based on the number of surface errors (e.g., misspellings of words, such as affliation vs. affiliation) and contextual errors (e.g., mistakes in subject verb agreement, such as “Our attributions about the causes of other people’s behavior *seems* to be…”) participants identified. Previous research has found that vigilance is associated with greater detection of more difficult (i.e., contextual) errors (Förster et al., 2003). The correlation between these two performance metrics was *r*(170) = 0.43, *p < 0*.001. Given the relatively strong correlation and given that the results do not differ as a function of performance metric, we present the results in the main text for the total number of identified errors metric and, for full transparency, present the detailed analyses for each individual metric (surface errors, complex errors) in the Supplementary material.

**Task-Related Variables (Skill, Enjoyment, and Familiarity)**. Participants then answered questions regarding the task (brainstorming or proofreading) they had just completed. Specifically, they responded to three questions designed to assess perceived task skill, enjoyment, and familiarity: How good are you at brainstorming (proofreading)? (1 = *very bad*, 6 = *very good*); How much did you enjoy the brainstorming (proofreading) task? (1 = *not at all,* 6 = *very much*); How often do you engage in brainstorming (proofreading)? (1 = *never,* 6 = *very often).*

### Results

#### Metamotivational knowledge of regulatory focus

**Figure 1:**
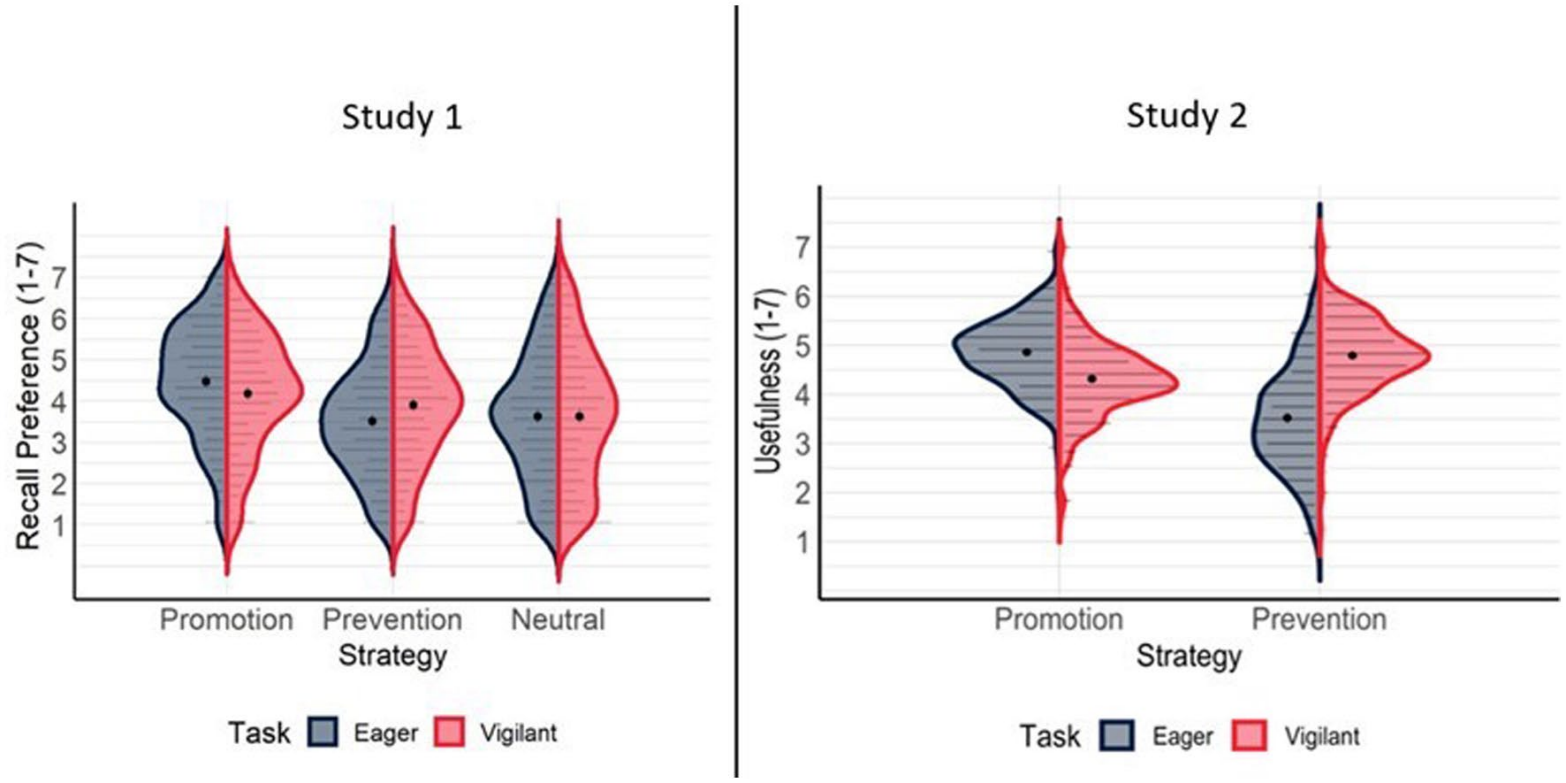
Recall Preference Ratings as a Function of Strategy and Task Type. Graph represents split violin plots with density distributions of recall preference ratings for each condition with individual data points (grey dots horizontally stacked within the shaded regions), descriptive means (black dots) and 95% confidence intervals (black error bars).

Participants’ metamotivational beliefs about regulatory focus were consistent with past work (Scholer and Miele, 2016). A significant task x recall activity interaction (Table 1) revealed that participants preferred promotion recall activities when anticipating eager relative to vigilant tasks. By contrast, participants preferred prevention recall activities when anticipating vigilant tasks relative to eager tasks. There was no difference in preference for neutral recall activities for eager vs. vigilant tasks. These comparisons reflect normative knowledge of task-motivation fit and also reveal substantial variability in this knowledge (Figure 1). In addition, participants demonstrated an overall preference for promotion activities, consistent with past work in this domain using these materials (Scholer and Miele, 2016; Nguyen et al., 2019)

**Table 1:** Repeated measures ANOVA: preference ratings for 2 (Task: Eager vs. Vigilant) × 3 (Strategy: Promotion vs. Prevention vs. Neutral).

Effect	*F*	*df*	*p*	η_p_^2^	*M* *(SD)*		
					Promotion	Prevention	Neutral
Recall Type	47.75	(1.55, 519.10)	<0.001	0.13	4.33 (2.65)_a_	3.71 (1.28)_b_	3.63 (1.45)_b_
					Eager	Vigilant	
Task Type	0.74	(1, 355)	0.391	0.002	3.87 (1.09)_a_	3.90 (1.13)_a_	
					Eager	Vigilant	
Task × Recall	31.06	(1.83, 614.48)	<0.001	0.09	4.48 (1.41)_a,1_	4.18 (1.41)_b,1_	(Promotion)
					3.51 (1.35)_a,2_	3.90 (1.44)_b,2_	(Prevention)
					3.63 (1.52)_a,2_	3.62 (1.63)_a,3_	(Neutral)

Significant differences between means are denoted with different letter subscript within rows and different numeric subscripts within columns (*p* < 0.05). Fractional df reflect Greenhouse–Geisser corrections for violations of sphericity for recall type [Mauchly’s W(2) = 0.71, *p* < 0.001] and the interaction [Mauchly’s W(2) = 0.91, *p* < 0.001]. Study did not moderate the interaction between task and recall activity, *F*(2, 611.95) = 1.23, *p* = 0.291.

#### Predicting overall task performance from total metamotivational knowledge

Next, we conducted a regression analysis to examine whether participants’ metamotivational knowledge predicted their performance on eager and vigilant tasks (i.e., brainstorming and proofreading), above and beyond other variables that may be related to task performance (i.e., task skill, enjoyment, and familiarity); see Table 2 for zero-order correlations.

**Table 2:** Zero-order correlations (Study 1).

	Total RF knowledge	Eager knowledge	Vigilant knowledge	Task skill	Task enjoyment	Task familiarity
Overall task performance (full sample; *N* = 336)	0.16 0.003	0.15 0.006	0.02 0.680	0.24 <0.001	0.25 <0.001	0.16 0.004
*Study 1a* (*n* = 169)	0.22 0.004	0.22 0.005	0.01 0.865	0.17 0.025	0.22 0.004	0.14 0.069
*Study 1b* (*n* = 167)	0.10 0.212	0.07 0.389	0.04 0.623	0.29 <0.001	0.27 <0.001	0.17 0.024
Proofreading performance (full sample; *N* = 170)	0.20 0.010	0.12 0.128	0.09 0.254	0.34 < 0.001	0.37 < 0.001	0.12 0.122
*Study 1a* (*n* = 84)	0.23 0.038	0.19 0.091	0.03 0.766	0.24 0.026	32 0.003	0.08 0.486
*Study 1b* (*n* = 86)	0.17 0.121	0.03 0.808	0.17 0.130	0.42 < 0.001	0.41 < 0.001	0.15 0.173
Brainstorming performance (full sample; *N* = 165)	0.13 0.094	0.20 0.011	−0.07 0.355	0.13 0.103	0.11 0.151	0.21 0.006
*Study 1a* (*n* = 85)	0.21 0.055	0.25 0.022	−0.03 0.810	0.11 0.337	0.10 0.368	0.23 0.035
*Study 1b* (*n* = 80)	0.01 0.925	0.12 0.275	−0.12 0.291	0.14 0.217	0.10 0.357	0.22 0.055
Brainstorming: # of ideas (Full sample; *N* = 165)	0.13 0.086	0.21 0.006	−0.09 0.269	0.13 0.097	0.10 0.213	0.20 0.012
*Study 1a* (*n* = 85)	0.21 0.055	0.27 0.014	−0.05 0.656	0.11 0.315	0.09 0.411	0.21 0.056
*Study 1b* (*n* = 80)	0.01 0.914	0.13 0.237	−0.13 0.255	0.14 0.225	0.08 0.486	0.20 0.078
Brainstorming: originality (full sample; *N* = 165)	−0.02 0.815	0.02 0.809	−0.05 0.543	0.08 0.338	0.11 0.160	0.19 0.017
*Study 1a* (*n* = 85)	−0.01 0.944	0.09 0.410	−0.14 0.213	0.11 0.302	0.03 0.769	0.20 0.064
*Study 1b* (*n* = 80)	−0.01 0.950	−0.03 0.826	0.02 0.868	0.06 0.605	0.19 0.092	0.17 0.123

To prepare the data, we standardized performance scores within each task condition—using the composite score for the brainstorming task and total number of errors detected for the proofreading task—and combined them to create a single index of overall task performance. We created an overall metamotivational knowledge index (*M =* 0.69, *SD* = 1.35) following the procedure: [promotion recall preferences for eager tasks– prevention recall preference for eager tasks] + [prevention recall preferences for vigilant tasks–promotion recall preferences for vigilant tasks] used by Scholer and Miele (2016). Higher numbers on this index indicate that participants demonstrate greater normatively accurate metamotivational beliefs (i.e., relatively greater preference for promotion activities when anticipating eager tasks and relatively greater preference for prevention activities when anticipating vigilant tasks). As can be observed in this index and consistent with the task x recall type interaction, on average participants had normatively accurate knowledge (*M* = 0.69 significantly greater than 0, *t*(335) = 9.37, *p* < 0.000, *d* = 1.35). To look at knowledge for eager and vigilant tasks separately, we also examined each component of the index separately (eager knowledge: promotion recall preferences for eager tasks– prevention recall preference for eager tasks; vigilant knowledge: prevention recall preferences for vigilant tasks– promotion recall preferences for vigilant tasks).

Continuous predictors were mean centered in all regression analyses in this study to allow for interpretation of the main effect of metamotivational knowledge in models with interaction terms. See Table 3 for descriptive statistics of Session 2 variables.

**Table 3:** Task performance descriptive statistics (Study 1).

Task	Performance metric	Performance			Task skill	Task enjoyment	Task frequency
		*M* *(SD)*	Min-Max	Skew	*M* *(SD)*	*M* *(SD)*	*M* *(SD)*
Brainstorming	Composite	5.44 (2.24)	0–12.5	0.94	3.46 (1.05)	3.56 (1.38)	3.26 (1.27)
	# of Ideas	7.82 (4.29)	0–21	1.08			
	Originality	3.06 (0.64)	0–5	−0.82			
Proofreading	# of Errors	12.38 (6.33)	0–34	0.31	3.61 (1.15)	3.46 (1.59)	3.44 (1.40)

We regressed participants’ performance scores on study (−1 = Study 1a, 1 = Study 1b), task type (−1 = brainstorming, 1 = proofreading), task skill, task enjoyment, task familiarity, total knowledge, and the interactions between total knowledge and both task type and study (see Table 4). As one might expect, task enjoyment and task skill were related to performance. Importantly, participants’ total metamotivational knowledge also predicted task performance. Notably, knowledge emerged as a significant predictor while controlling for skill, enjoyment, and frequency of engaging in that type of task. Greater total knowledge was related to enhanced performance on both tasks (i.e., there was no task type × knowledge interaction). Although there was no task type × knowledge interaction on overall performance, for full transparency, we also

**Table 4:** Regression analyses predicting task performance from total knowledge, controlling for study and task type, skill, enjoyment, and familiarity (Study 1).

Predictors	*B*	SE	β	*t*	*p*	95% CI
Intercept	−0.25	0.17		−1.99	0.047	[−0.50, 0.003]
Total Knowledge	0.11	0.04	0.15	2.70	0.007	[0.03, 0.18]
Task Type	0.02	0.05	0.02	0.34	0.737	[−0.08, 0.12]
Study	−0.03	0.05	−0.03	−0.59	0.556	[−0.13, 0.07]
Task Skill	0.11	0.06	0.12	1.83	0.069	[−0.01, 0.23]
Task Enjoyment	0.09	0.04	0.14	2.17	0.031	[0.01, 0.17]
Task Familiarity	0.04	0.05	0.05	0.81	0.421	[−0.05, 0.13]
Knowledge*Task Type	0.06	0.04	0.08	1.41	0.159	[−0.02, 0.14]
Knowledge*Study	−0.08	0.04	−0.11	−1.96	0.051	[−0.16, 0.003]

report the results of separate regression analyses for each performance metric (brainstorming: composite, number of alternatives, originality; proofreading: composite, surface errors, contextual errors) in the Supplementary material. There was a marginal interaction between knowledge and study, indicating that the effect of knowledge on performance was likely moderated by study (as also indicated by the zero-order correlations in Table 2). Knowledge emerged as a significant predictor of task performance in Study 1a (*b* = 0.19, *p* = 0.001), but not in Study 1b (*b* = 0.03, *p* = 0.640; see Supplementary material for details).

#### How do eager and vigilant knowledge relate to performance?

Next, we separated the total knowledge index into its two component indices and conducted a more focused analysis examining whether eager and vigilant knowledge predict overall task performance, using the composite performance metrics from the previous analysis. These analyses provide insight into whether the associations between eager and vigilant knowledge and performance vary as a function of task affordance (i.e., whether vigilant knowledge is more closely associated with performance on the vigilant proofreading task).

We first regressed task performance on eager knowledge, vigilant knowledge, task type, study, task skill, task enjoyment, task familiarity, the interactions between both types of knowledge and task type, and the interactions between both types of knowledge and study (see Table 5a). Results revealed that eager knowledge predicted overall task performance. That is, eager knowledge was related to better performance on both the brainstorming and proofreading tasks. However, there was also a marginal interaction between study and eager knowledge that paralleled the pattern found with total knowledge. That is, eager knowledge emerged as a significant predictor of performance in Study 1a, but not Study 1b (see Supplementary Table S11 for more details).

**Table 5:** Regression analyses predicting task performance from eager and vigilant knowledge, controlling for study and task type, skill, enjoyment, and familiarity (Study 1).

Dependent variable	Predictors	*b*	SE	*β*	*t*	*p*	95% CI
(a) Overall task performance	Intercept	−0.26	0.13		−2.07	0.039	[−0.51, −0.01]
	Eager Knowledge	0.12	0.05	0.16	2.67	0.008	[0.03, 0.22]
	Vigilant Knowledge	0.07	0.05	0.08	1.39	0.166	[−0.03, 0.17]
	Task Type	0.01	0.05	0.01	0.25	0.803	[−0.09, 0.12]
	Study	−0.03	0.05	−0.03	−0.60	0.547	[−0.13, 0.07]
	Task Skill	0.10	0.06	0.12	1.70	0.090	[−0.02, 0.22]
	Task Enjoyment	0.10	0.04	0.15	2.26	0.025	[0.01, 0.18]
	Task Familiarity	0.03	0.05	0.05	0.73	0.464	[−0.06, 0.12]
	Eager*Task Type	0.03	0.05	0.04	0.62	0.539	[−0.06, 0.12]
	Vigilant*Task Type	0.11	0.05	0.12	2.07	0.039	[0.01, 0.21]
	Eager*Study	−0.09	0.05	−0.12	−1.96	0.051	[−0.18, 0.004]
	Vigilant*Study	−0.05	0.05	−0.06	−1.07	0.286	[−0.15, 0.05]
(b) Proofreading (number of errors detected)	Intercept	9.61	1.01		9.78	<0.001	[7.61, 11.61]
	Eager Knowledge	0.87	0.39	0.18	2.23	0.029	[0,10 1.64]
	Vigilant Knowledge	1.10	0.42	0.21	2.63	0.010	[0.27, 1.92]
	Study	−0.45	0.45	−0.07	−1.00	0.324	[−1.34, 0.45]
	Task Skill	1.11	0.49	0.20	225	0.026	[0.14, 2.08]
	Task Enjoyment	1.01	0.34	0.26	2.98	0.003	[0.34, 1.68]
	Task Familiarity	−0.31	0.35	−0.07	−0.89	0.376	[−1.01, 0.39]
	Eager*Study	−0.82	0.39	−0.17	−2.10	0.037	[−1.59, −0.05]
	Vigilant*Study	−0.46	0.42	−0.09	−1.11	0.268	[−1.29, 0.36]
(c) Brainstorming (composite score)	Intercept	5.58	0.46		12.21	<0.001	[4.68, 6.48]
	Eager Knowledge	0.29	0.15	0.16	1.86	0.065	[−0.02, 0.59]
	Vigilant Knowledge	−0.01	0.18	−0.003	−0.04	0.968	[−0.37, 0.35]
	Study	−0.23	0.17	−0.10	−1.31	0.192	[−0.57, 0.12]
	Task Skill	−0.02	0.21	−0.01	−0.12	0.908	[−0.44, 0.39]
	Task Enjoyment	−0.06	0.15	−0.04	−0.37	0.710	[−0.36, 0.25]
	Task Familiarity	0.46	0.18	0.25	2.61	0.010	[0.11, 0.80]
	Eager*Study	−0.15	0.15	−0.09	−0.98	0.328	[−0.45, 0.15]
	Vigilant*Study	−0.15	0.18	−0.07	−0.85	0.398	[−0.50, 0.20]

Results revealed that task type moderated the effect of vigilant knowledge on performance. To explore this interaction, we conducted additional regression analyses with brainstorming performance and proofreading performance as the outcomes. Vigilant knowledge was related to performance on the proofreading task (see Table 5b) but not the brainstorming task (see Table 5c). This effect was consistent across Studies 1a and 1b. As can be seen in Table 5c, participants’ eager knowledge also predicted proofreading performance, but this effect was significantly moderated by study. Eager knowledge emerged as a significant predictor of proofreading performance in Study 1a (*b* = 1.72, *p* = 0.005), but not in Study 1b (*b* = 0.05, *p* = 0.927; see Supplementary material for details).

To further examine if these knowledge components were more strongly related to performance on particular metrics, we examined whether eager knowledge and vigilant knowledge were associated with each metric of task performance (brainstorming: number of ideas; brainstorming: originality; proofreading: surface errors; and proofreading: complex errors). The results did not substantially differ for the two metrics on the proofreading task. We present the detailed analyses in the Supplementary material. Eager, but not vigilant, knowledge predicted the total number of ideas generated in the brainstorming task (Table 6). Knowledge did not predict the originality of ideas (Table 7). Study did not moderate these effects.

**Table 6:** Regression analyses predicting brainstorming performance (total number of ideas) from eager and vigilant knowledge, controlling for study and task skill, enjoyment, and familiarity.

Predictors	*B*	SE	β	*t*	*p*	95% CI
Intercept	8.23	0.87		9.42	<0.001	[6.50, 9.99]
Eager Knowledge	0.59	0.29	0.17	1.99	0.048	[0.01, 1.17]
Vigilant Knowledge	−0.05	0.35	−0.01	−0.14	0.890	[−0.74, 0.64]
Study	−0.50	0.33	−0.12	−1.52	0.132	[−1.16, 0.15]
Task Skill	0.04	0.40	0.01	0.10	0.918	[−0.75, 0.83]
Task Enjoyment	−0.16	0.29	−0.05	−0.55	0.586	[−0.74, 0.42]
Task Familiarity	0.79	0.34	0.23	2.34	0.021	[0.12, 1.46]
Eager*Study	−0.27	0.29	−0.08	−0.93	0.353	[−0.85, 0.31]
Vigilant*Study	−0.27	0.34	−0.07	−0.79	0.433	[−0.94, 0.41]

**Table 7:** Regression analyses predicting brainstorming performance (originality) from eager and vigilant knowledge, controlling for study and task skill, enjoyment, and familiarity.

Predictors	*B*	SE	*β*	*t*	*p*	95% CI
Intercept	2.96	0.13		22.15	<0.001	[2.69, 3.22]
Eager Knowledge	−0.003	0.05	−0.01	−0.06	0.950	[−0.09, 0.09]
Vigilant Knowledge	−0.03	0.05	−0.05	−0.57	0.569	[−0.14, 0.08]
Study	0.06	0.05	0.09	1.13	0.261	[−0.04, 0.16]
Task Skill	−0.04	0.06	−0.06	−0.62	0.539	[−0.16, 0.08]
Task Enjoyment	0.04	0.05	0.08	0.82	0.415	[−0.05, 0.13]
Task Familiarity	0.10	0.05	0.18	1.85	0.066	[−0.01, 1.00]
Eager*Study	−0.04	0.05	−0.07	−0.78	0.435	[−0.12, 0.05]
Vigilant*Study	0.03	0.05	0.05	0.65	0.520	[−0.07, 0.14]

**Figure 2:**
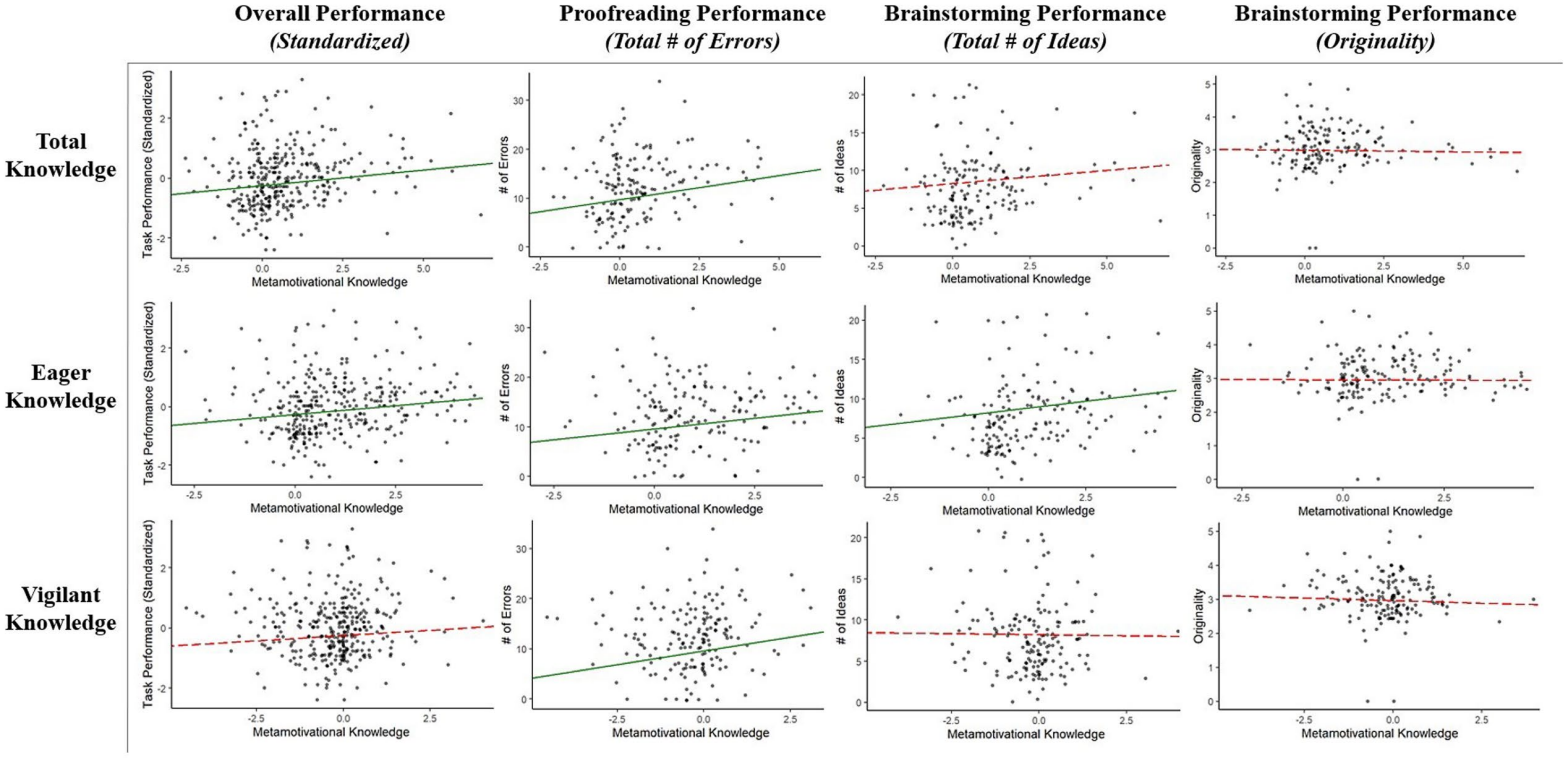
Scatterplots with Regression Lines for Total, Eager, and Vigilant Knowledge Predicting Performance (Overall [Standardized]; Proofreading [# of Errors]; Brainstorming [# of Ideas], and Brainstorming [Originality]). Green, solid lines reflect a significant relationship between knowledge and performance; red, dashed lines reflect a non-significant relationship. Overall Performance = performance collapsed and standardized across tasks; Proofreading Performance = total # of errors detected in proofreading task; Brainstorming Performance (Total # of Ideas) = total number of ideas generated in brainstorming task; Brainstorming Performance (Originality) = average originality score of generated ideas in brainstorming task.

In sum, total metamotivational knowledge was a significant predictor of overall performance and proofreading performance but did not significantly predict brainstorming performance on either metric (number of ideas or originality ratings). Eager knowledge also significantly predicted overall performance and proofreading performance; it was also a significant predictor of the total number of ideas generated for the brainstorming task (but not originality ratings). Vigilant knowledge was only significantly related to proofreading performance. See Figure 2 for a visual summary of these findings.

### Discussion

Study 1 provides an initial examination of whether metamotivational knowledge of regulatory focus task-motivation fit predicts performance in single-shot lab tasks. There were several notable findings. First, metamotivational knowledge was related to task performance in the combined sample. Second, eager knowledge was associated with total number of ideas generated on the brainstorming task, but not the coded originality of ideas, suggesting that eager knowledge was not equally related to these components of creativity. Third, eager knowledge was related to performance on the proofreading task and on brainstorming idea generation, while vigilant knowledge was related to performance only on the proofreading task. Fourth, the relation between knowledge and performance was consistently observed in Study 1a, but not 1b.

Although metamotivational knowledge was related to overall performance on the brainstorming task, this effect was driven by the relation of knowledge to the number of ideas generated rather than to the coded originality of ideas. Prior work on regulatory focus has shown that promotion motivation is related both to the number of ideas that people are likely to generate and the originality of ideas (Friedman and Förster, 2001). One might argue that the task instructions used in the current study placed greater emphasis on the number of ideas compared to the originality of ideas. It is also possible that the knowledge assessment may have better detected knowledge that supports the generation of ideas on relevant tasks, rather than knowledge that supports uniqueness—a possibility to explore in future research.

In addition, the relation between the knowledge components and task performance was not symmetric. Eager knowledge was related to performance on both tasks, whereas vigilant knowledge was related to performance only on the proofreading task. One possibility is that understanding when a given motivation is *not* useful plays an important role in understanding when it *is* useful (Nguyen et al., 2019). It is also very likely that these tasks are not process pure, despite having a dominant motivational affordance (see also Förster et al., 2003). In particular, there were two features of the proofreading task that might have pulled for eager affordances. The instructions indicated that participants should complete the task as quickly as possible and identified errors were, by default, highlighted in green (rather than crossed out). This presentation may have inadvertently made the finding of errors something that could be perceived both as eliminating a loss (taking away the error) and adding a gain (emphasizing that another error has been successfully identified).

Lastly, we want to return to the finding that the relation between metamotivational knowledge and performance was observed in Study 1a, but not Study 1b. As detailed in the Supplementary material, there were no clear differences between the samples that can easily explain this unpredicted difference. Rather, we think this may be due to both the nature of the performance assessment—a single-shot, 3-min task—and the dynamics of how metamotivational knowledge gets translated into action. This variability in the apparent robustness of the effect may be conceptually meaningful for understanding when and why metamotivational knowledge will be directly associated with performance.

There are a number of different ways that individuals may deploy their knowledge that were not available to participants in this single-shot performance opportunity where the task was assigned. In many contexts, people have the option to regulate goal performance not only by selecting a motivational strategy for a given task (e.g., “I’ll think about what I can gain by doing well on this task!”), but by selecting a task based on a current motivational state (“I’m feeling really eager, so I’ll start with the creativity task!”). In this study, the only way to create task-motivation fit was to change or sustain a desired motivational orientation via the strategies one used. Not only were participants constrained in this way, but they also may not have had the same strategies available to them that they typically would use spontaneously or may not have been able to quickly generate strategies in this unfamiliar context. Thus, when performance is a brief, single-shot opportunity such as the paradigm in Study 1, there is only one chance for these factors to align such that a direct association between knowledge and performance is observed.

Many self-regulatory situations, however, provide multiple opportunities for people to pursue their goals more or less effectively. For instance, students in a college course have many occasions that contribute to their learning and performance. They can take notes more or less effectively, read the text more or less effectively, study for exams more or less effectively, and show up to class (or not). They can select tasks based on their current motivational state, or they can attempt to change their current motivational state to meet current task demands. They can experiment with the effectiveness of strategies across learning opportunities. In other words, situations that afford multiple opportunities for the application of metamotivational knowledge may allow for the observation of a stronger direct link between knowledge and performance. We explore this in Study 2.

## Study 2

Study 2 examined whether metamotivational knowledge of regulatory focus task-motivation fit was related to academic performance (in an introductory psychology course), a situation in which there are many opportunities for knowledge to shape outcomes. Given that academic motivation and past academic success strongly predict academic performance (Robbins et al., 2004), Study 2 also tested whether metamotivational knowledge predicts performance above and beyond these traditional predictors of grades.

Because final grades in a course are comprised of many tasks that likely afford both eagerness and vigilance, we conducted a pilot study (final *N* = 109) that assessed students’ perceptions of the usefulness of promotion and prevention focus (1 = *extremely unhelpful*, 7 = *extremely helpful*; see Supplementary material for details) for the overall course and for each graded component of the course: exams (3, non-cumulative, multiple choice); section points (class participation); research participation; and a final reflection paper. Results revealed that participants viewed PSYCH 1100 as a course that benefitted more from engaging with a promotion focus (*M =* 6.05, *SD* = 1.15) than with a prevention focus (*M =* 5.55, *SD* = 1.34), *t*(108) = 3.22, *p* = 0.002. However, both responses were significantly above the midpoint (4), *promotion*: *t*(108) = 18.65, *p* < 0.001; *prevention*: *t*(108) = 12.07, *p* < 0.001, suggesting that students perceived both promotion and prevention motivation as beneficial for course performance.

### Materials and methods

#### Participants

We recruited 592 students in an introductory psychology course (PSYCH 1100) from a large Midwestern university (*M*_age_ = 19.25, *SD*_age_ = 2.19; 279 women, 306 men, 7 did not report gender; 64.4% White/European American, 18.6% Asian American, 6.3% mixed racial/ethnic identity, 4.9% Black/African American, 3.0% Hispanic/Latinx, 1.2% Middle Eastern; 1.7% did not report) for a larger online study^4^, for which they received partial course credit. Study 2 was conducted across two semesters (Study 2a: weeks 12–14 of the semester; Study 2b: weeks 11–15 of the semester); as in Study 1, we present results combined across semesters. Measures unrelated to the current research questions are described in the Supplementary material. This study was reviewed and approved by The Ohio State University Institutional Review Board, and participants provided their written informed consent to participate in the study.

**Exclusion criteria and sensitivity analyses.** Consistent with research practices for online studies for the lab that conducted Study 2, we made *a priori* decisions to exclude a subset of participants across analyses. We excluded participants who reported not paying attention (i.e., reported being “very” or “extremely” distracted or taking the study “not at all” or “a little” seriously; *n* = 122) and non-native English speakers (*n* = 84). Additional exclusions were necessary for a subset of participants who did not have complete data—i.e., those who did not consent to share their grades (*n* = 32), those whose academic records we could not retrieve (*n* = 5), those who did not report a high school GPA (*n* = 38). Moreover, inspection of the data revealed that two participants had unusual degrees of missing data (i.e., > 50% of the knowledge assessment was left blank). Data were analyzed with and without these participants, and we report the results of the former in the Supplementary material and the latter in the main text. After exclusions^5^, we had a final *N* = 368 (Study 2a, *n* = 206, Study 2b, *n* = 162; *M*_age_ = 19.10, *SD*_age_ = 1.63; 186 women, 180 men, 2 did not report; 77.4% White/European American, 7.3% Asian American, 7.3% mixed racial/ethnic identity, 4.6% Black/African American, 2.4% Hispanic/Latinx, 0.5% Middle Eastern; 0.3% did not report).^6^ A sensitivity analysis revealed that our final *N* = 368 provided 90% power to detect an effect as small as *f*^2^ = 0.03 —a linear multiple regression analysis (two-tailed, 9 predictors).

#### Materials

**Metamotivational knowledge assessment**. Participants completed a knowledge assessment similar to the one used in Study 1. This measure did not include neutral recall activities given that those are not included in the calculation of normative accuracy. Participants rated the usefulness of recall activities for task performance (1 = *extremely unhelpful*, 7 = *extremely helpful*). The number of tasks were increased, such that the assessment included six tasks (3 eager, 3 vigilant) and eight recall activities (4 promotion focus, 4 prevention focus), for a total of 48 randomly presented task and recall activity pairs; full materials are in the Supplementary material.

**Academic motivation.** Participants then rated their motivation to do well in PSYCH 1100 on three items, which were averaged to create a composite score: How [important is it for you/valuable would it be for you/motivated are you] to do well in PSYCH 1100? (α = 0.85; 1 = *not at all*, 7 = *extremely*).

**Academic self-concept.** Participants also rated their history of success in the academic domain on three items (Fishbach et al., 2003), which were averaged to create composite score: How successful are you at studying effectively? How difficult is it for you to prepare adequately for your exams? (reverse-coded) How successful are you at getting good grades? (α = 0.67; 1 = *not at all*, 7 = *extremely*).

**High school GPA.** As another measure of past academic success, we asked participants to report their unweighted high school GPA.

**Demographics and final measures.** Participants reported how distracted they were during the study and how seriously they took the study (1 = *not at all*, 2 = *slightly*, 3 = *somewhat*, 4 = *very,* 5 = *extremely*). Participants also reported their gender, age, and major. Final course grades were obtained at the end of the semester through administrative help from the university’s registrar office.

### Results

#### Metamotivational knowledge of regulatory focus

Participants’ metamotivational beliefs about regulatory focus were consistent with past work (Scholer and Miele, 2016). A significant task x recall activity interaction (see Table 8) revealed that participants rated promotion recall activities as more useful for eager relative to vigilant tasks. By contrast, participants rated prevention recall activities as more useful for vigilant relative to eager tasks. These comparisons reflect knowledge of task-motivation fit and also reveal substantial variability in this knowledge (see Figure 1). In addition, participants rated promotion activities as most useful, consistent with past work in this domain using these materials (Scholer and Miele, 2016; Nguyen et al., 2019).

**Table 8:** Repeated measures ANOVA: preference ratings for 2 (Task: Eager vs. Vigilant) × 3 (Strategy: Promotion vs. Prevention vs. Neutral).

Effect	*F*	df	*p*	*η*_p_^2^	*M* *(SD)*		
					Promotion	Prevention	
Recall type	187.32	(1, 367)	<0.001	0.34	4.59 (0.68)_a_	4.16 (0.75)_b_	
					Eager	Vigilant	
Task type	168.02	(1, 367)	<0.001	0.31	4.19 (0.68)_a_	4.56 (0.67)_b_	
					Eager	Vigilant	
Task × Recall	497.55	(1, 367)	<0.001	0.58	4.86 (0.72)_a,1_	4.32 (0.80)_b,1_	(Promotion)
					3.52 (1.10)_a,2_	4.79 (0.77)_b,2_	(Prevention)

Significant differences between means are denoted with different letter subscript within rows and different numeric subscripts within columns (*p* < 0.05). Study did not moderate the interaction between task and recall activity, *F*(1, 366) = 0.33, *p* = 0.565, η_p_^2^ = 0.001.

#### Predicting grades from knowledge of task-motivation fit

Next, we conducted a regression analysis to examine whether individual differences in knowledge of how to create regulatory focus task-motivation fit predict students’ final course grade, above and beyond traditional correlates of grades and other covariates (see Table 9 for zero-order correlations). To prepare the data, we converted letter grades to a 4-point scale (A = 4.0, A- = 3.7, B + =3.4, etc.; *M* = 3.40, *SD* *= 0*.77). We also created the same overall metamotivational knowledge index (*M =* 1.82, *SD* = 1.56) as in Study 1. Continuous predictors were standardized in all regression analyses to facilitate meaningful interpretations in the predicted change in grades.

**Table 9:** Zero-order correlations for Study 2.

	1	2	3	4	5	6	7	8	9
1. Final Grade in PSYCH 1100	-								
2. Total Knowledge	0.31***	-							
3. Eager Knowledge	0.28***	0.86***	-						
4. Vigilant Knowledge	0.19***	0.72***	0.26***	-					
5. High School GPA	0.33***	0.16**	0.16**	0.08	-				
6. Academic Motivation	0.15**	0.03	0.03	−0.01	0.11*	-			
7. Academic Self-Concept	0.41***	0.12*	0.08	0.12*	0.19***	0.17**	-		
8. Gender (higher = female)	0.13*	−0.01	0.06	−0.09	0.16**	0.17**	−0.001	-	
9. Age	−0.23***	0.01	0.02	−0.01	−0.35***	−0.16**	−0.07	−0.21***	-
10. Major (higher = psych major)	−0.004	−0.04	−0.02	−0.05	0.01	0.15**	−0.04	0.11*	−0.04

*p < 0.05, **p < 0.01, ***p < 0.001.

We regressed grades on total knowledge, high school GPA, academic motivation, academic self-concept, gender (−0.5 = male, 0.5 = female or unidentified), age, major (−0.5 = other major, 0.5 = psychology), study (−0.5 = Study 2a, 0.5 = Study 2b), and total knowledge x study (see Table 10). High school GPA, academic self-concept, and age significantly predicted grades. Importantly though, as expected, students’ knowledge of how to create task-motivation fit also significantly predicted their final grades. In fact, students’ metamotivational knowledge was the second strongest predictor of course grades after self-reported academic self-concept.

**Table 10:** Regression analysis predicting final grades—total knowledge (Study 2).

Predictors	*b*	SE	*β*	*t*	*p*	95% CI
Intercept	3.40	0.06		62.07	<0.001	[3.29, 3.50]
Total Knowledge	0.18	0.04	0.24	5.31	<0.001	[0.12, 0.25]
High School GPA	0.13	0.04	0.17	3.53	<0.001	[0.06, 0.21]
Academic Motivation	0.03	0.04	0.04	0.92	0.357	[−0.04, 0.10]
Academic Self-Concept	0.25	0.04	0.33	7.14	<0.001	[0.18, 0.32]
Gender	0.11	0.07	0.07	1.49	0.138	[−0.03, 0.25]
Age	−0.10	0.04	−0.12	−2.55	0.011	[−0.17, −0.02]
Major	−0.003	0.11	−0.001	−0.03	0.978	[−0.22, 0.21]
Study	−0.02	0.07	−0.01	−0.31	0.760	[−0.16, 0.11]
Total Knowledge * Study	−0.04	0.07	−0.03	−0.60	0.549	[−0.18, 0.09]

Next, we examined what kind of knowledge predicts grades. To do so, we separated the total knowledge index into two indices as in Study 1. We regressed grades on eager knowledge, vigilant knowledge, high school GPA, academic motivation, academic self-concept, gender (−0.5 = male, 0.5 = female or unidentified), age, major (−0.5 = other major, 0.5 = psychology), study (−0.5 = Study 2a, 0.5 = Study 2b), eager knowledge x study, and vigilant knowledge x study (see Table 11). Results revealed that both eager and vigilant knowledge predicted grades.

**Table 11:** Regression analysis predicting final grades—eager and vigilant knowledge (Study 2).

Predictors	*b*	SE	*β*	*t*	*p*	95% CI
Intercept	3.40	0.06		61.80	<0.001	[3.29, 3.50]
Eager Knowledge	0.16	0.04	0.21	4.31	<0.001	[0.08, 0.23]
Vigilant Knowledge	0.07	0.04	0.09	1.97	0.050	[0.00002, 0.14]
High School GPA	0.13	0.04	0.17	3.47	0.001	[0.06, 0.21]
Academic Motivation	0.03	0.04	0.04	0.90	0.368	[−0.04, 0.10]
Academic Self-Concept	0.25	0.04	0.33	7.17	<0.001	[0.18, 0.32]
Gender	0.10	0.07	0.06	1.37	0.171	[−0.04, 0.24]
Age	−0.10	0.04	−0.13	−2.59	0.010	[−0.17, −0.02]
Major	−0.004	0.11	−0.002	−0.04	0.971	[−0.22, 0.21]
Study	−0.02	0.07	−0.01	−0.27	0.788	[−0.15, 0.12]
Eager knowledge * Study	−0.03	0.07	−0.02	−0.39	0.698	[−0.17, 0.11]
Vigilant knowledge * Study	−0.02	0.07	−0.01	−0.31	0.757	[−0.16, 0.12]

### Discussion

Study 2 demonstrated that students’ understanding of how to create task-motivation fit predicts their academic performance in an introductory psychology course, above and beyond traditional predictors of academic success. This work extended Study 1 by demonstrating the relation between metamotivational knowledge and performance in a more naturalistic and consequential setting that unfolds over time. Interestingly, results showed that both eager knowledge and vigilant knowledge contributed to predicting final course grades, although the former was a stronger predictor. Given the perceptions of motivational affordances found in the pilot study—i.e., that participants consider the course to benefit more from engaging with a promotion focus than with a prevention focus—it is not necessarily surprising that eager (vs. vigilant) knowledge was more strongly related to grades. However, as noted in the study introduction, students perceived that both promotion and prevention focus were useful in the context of this course. This highlights an important dynamic in goal pursuit with respect to task-motivation fit; sometimes the *same* goal pursuit can have subgoals or components that differ in the extent to which they afford promotion versus prevention motivation. Thus, metamotivational flexibility is not only relevant when switching motivational strategies as a function of pursuing completely different goals, but also can be about switching motivational strategies in the midst of pursuing the same goal.

## General discussion

The current studies provide initial evidence that the normative accuracy of people’s metamotivational knowledge of regulatory focus task-motivation fit predicts performance on brief, single-shot tasks (Study 1) and in a more consequential setting that involves goal pursuit unfolding over time (Study 2). Although prior work has shown that people have, on average, normatively accurate metamotivational knowledge of regulatory focus task-motivation fit (Scholer and Miele, 2016; Nguyen et al., 2019), these studies are the first to test the relation between this knowledge and performance. Notably, the relationship between knowledge and performance was particularly robust in Study 2; students’ understanding of how to create task-motivation fit predicted their academic performance, above and beyond traditional predictors of academic success. A meta-analysis of the raw correlations from all four samples revealed that higher metamotivational knowledge was positively related to performance, $\bar{r\;}$ = 0.24, 95% CI [0.07, 0.41] (see Supplementary material for details). Taken together, these studies suggest that having metamotivational knowledge of regulatory focus task-motivation fit may be one factor that influences performance, though as discussed earlier and in more detail below, the variability in the robustness of the effect suggests that there are likely many factors that influence this relationship.

### A metamotivational approach to improving performance

The current work advances our understanding of factors that may contribute to successful goal performance, adding to a growing literature that suggests that people’s beliefs and knowledge about the ways that motivation and cognition works can be impactful in goal pursuit. In particular, the burgeoning area of research on metamotivation suggests new approaches to understanding when and why performance may soar or suffer, and identifies novel targets of intervention. An individual who believes in the universal efficacy of prevention strategies will face a different set of performance challenges and may require a different kind of intervention than an individual who believes in the universal efficacy of promotion strategies. Because this approach emphasizes the importance of investigating people’s beliefs and knowledge about how motivation works, it suggests the importance of targeting knowledge, not only regarding the various strategies that can be used to induce or sustain a motivational state, but also the knowledge of when to deploy particular strategies based on task demands.

Previous interventions designed to increase performance have often focused on developing general capacities (e.g., self-control, executive functioning) known to support performance or by changing situational factors (e.g., reducing temptations in the immediate environment) that influence goal pursuit. These approaches can certainly be effective; for example, interventions designed to enhance participants’ self-regulatory skills have led to increased physical exercise and healthy eating (Fleig et al., 2011) and weight loss success (Frie et al., 2020). One limitation of these approaches, however, is that they often, at least implicitly, imply a “one size fits all” approach to motivation, suggesting that a particular regulatory strategy will generally be beneficial (“the fallacy of uniform efficacy”; Bonanno and Burton, 2013). By contrast, a metamotivational intervention approach could be built around improving people’s knowledge of “if…then” contingencies pertaining to motivational effectiveness. One strength of this approach is that it recognizes that an individual’s beliefs may lead to a unique set of self-regulatory obstacles standing in their way of successful goal performance, and therefore interventions may better equip them to flexibly navigate these challenges (Miele et al., 2020). For this reason, metamotivation is distinct from simply being “good” at self-regulation and offers researchers a valuable approach to help people achieve their goals.

#### Normative vs. idiographic knowledge

The current studies suggest that having *normatively* accurate metamotivational knowledge of the benefits of regulatory focus task-motivation fit is related to performance. However, research has yet to explore how the accuracy of beliefs and knowledge regarding one’s own idiographic motivational experiences might relate to performance. People may develop these personal task-motivation associations in several ways, and these may sometimes diverge from normative effects. For instance, it is possible that an individual with an extremely strong chronic promotion focus may find it especially difficult to initiate and sustain prevention motivation. Although on average people perform better on vigilant tasks when in a prevention state (Förster et al., 2003), this particular individual may have learned that vigilant strategies are generally not effective for them. This idiographic accuracy may play an especially important role in situations where there is no one qualitative motivational state that can clearly be identified as superior. Further exploring the role of both normative and idiographic accuracy of metamotivational knowledge is an exciting direction for future research.

#### Factors influencing the implementation of metamotivational knowledge

As discussed at length in the Study 1 discussion, there are many factors that may influence the likelihood that metamotivational knowledge gets translated into performance in a given moment. If performance is aggregated across many such moments the relationship between metamotivational knowledge and performance is likely to be more robust, which is consistent with the effect documented in Study 2. However, if performance is based on a single opportunity, we suspect that knowledge will not always lead to better performance, consistent with the variability in the effect documented in Study 1. Some specific factors we believe will be important to examine in future work include self-knowledge (i.e., awareness of one’s own motivational states and the particular strategies that would be most effective for oneself), the repertoire of strategies available in a given situation, and lay beliefs about the malleability of motivation.

#### Motivation regulation in the broader context of self-regulation

The study of metamotivation focuses on motivation as the target of regulation (Scholer et al., 2018), but, as has been discussed extensively elsewhere, the means by which individuals regulate motivation could involve multiple strategies involving altering cognitions, emotions, or behaviors (Miele and Scholer, 2018). Further, in the process of goal pursuit, individuals engage in additional meta-regulatory processes that target emotions (Ford and Tamir, 2012), cognitions (Nelson and Narens, 1990), and behavior. For example, individuals may have beliefs about emotions and motivations that are synergistic (e.g., recognizing that happiness is a signal of promotion-relevant success) or at odds (e.g., holding an emotion regulation goal to be relaxed while trying to induce prevention motivation for a vigilant task). An intriguing avenue for future work will be to continue to examine how these meta-regulatory processes intersect to affect performance (see also Webster and Hadwin, 2015).

### Limitations

One limitation of the present work is the specificity of the measure used to assess metamotivational knowledge. Scoring high on the accuracy index indicates that people recognize the normative benefits (or drawbacks) of the task-strategy pairings being presented to them. While assessing knowledge in this way is useful, it is also constrained in that it imposes a relatively narrow definition of what it means to have accurate metamotivational knowledge. That is, the current knowledge assessment fails to capture several potentially important factors, including whether people are able to spontaneously generate strategies in the moment, and whether their own prior experiences conflict with these normative performance standards. Therefore, the ability to fully understand the role of knowledge in performance may require further development and validation of different types of diagnostic measures of metamotivational knowledge.

Furthermore, we see the current analyses as a preliminary approach to exploring psychometrics and think that considering other approaches to measuring metamotivational knowledge may improve the robustness of the measure. Specifically, the assessments in the current research measured participants’ knowledge about different recall activities using separate bipolar scales. For example, participants rated the usefulness of a promotion recall activity for an eager task (1 = extremely unhelpful, 7 = extremely helpful) and they also rated the usefulness of a prevention recall activity for an eager task (1 = extremely unhelpful, 7 = extremely helpful). Metamotivation research in the construal level theory domain has taken a different measurement approach using the relative preference for motivational strategies on a single scale (Nguyen et al., 2022). Future research should investigate the implications of structuring the metamotivational knowledge assessment in different ways.

Another limitation of the current work is the relatively artificial nature of the lab performance tasks in Study 1. The benefit of using the brainstorming and proofreading tasks is that prior work has shown that these specific tasks have eager and vigilant motivational affordances, respectively (Friedman and Förster, 2001; Förster et al., 2003), and thus they provide a relatively “clean” test of whether metamotivational knowledge matters in this domain. However, a significant downside of this paradigm is that the tasks are presumably low-stakes to most participants (there were no clear incentives for performing well). Thus, the ability to generalize from these tasks to richer, more complex real-world contexts is constrained. Although the examination of course grades in Study 2 addresses some of these concerns, this is a limitation that needs to be further explored. Furthermore, Study 2 focuses on one course (introductory psychology) and therefore is unable to generalize to other courses. Future research should examine these effects in an array of real-world contexts, both inside and outside the classroom.

The present research is also limited in its ability to generalize beyond WEIRD participants (Western, Educated, Industrialized, Rich, and Democratic; Henrich et al., 2010). Both samples were comprised of university students from Western cultures (i.e., Canada and the United States). Prior work has revealed cross-cultural similarities in metamotivational knowledge with Japanese participants (Nguyen et al., 2019), yet this research also relied on relatively educated samples. Thus, it will be important in future work to continue to examine metamotivation in more diverse samples, and to examine the types of experiences that may contribute to the development and implementation of specific motivational beliefs.

Finally, it is important acknowledge the correlational nature of this work. It is possible that participants who came into the studies as high achievers may have had higher levels of metamotivational knowledge; thus, no strong causal claims can be made about the effects of knowledge on performance, even when controlling for prior performance. Future research should investigate the development of knowledge and test interventions designed to teach people about these normative effects of motivation found in the literature, thereby allowing for a better understanding of the potential causal effect of metamotivational knowledge on performance. Similarly, to understand how knowledge enhances performance, another important direction for future work is to examine the specific ways in which people monitor and control their motivation while pursuing a task or goal (see Miele and Scholer, 2018).

## Conclusion

The present research provides initial evidence that metamotivational knowledge of regulatory focus task-motivation fit predicts performance. Given that those who can effectively manage and pursue their goals experience benefits in a number of domains—including higher life satisfaction, better psychological adjustment, better achievement in work and academic domains, and fewer health problems (see Tangney et al., 2004)—it will be interesting to explore whether and why accurate metamotivational knowledge may be related to these outcomes as well. By examining the role of metamotivational knowledge in goal-relevant task-performance, this research offers new insights for goal-pursuit and self-regulatory success. The more we understand about individuals’ beliefs and knowledge of motivation, the more we can think about where and when people tend to go right or wrong in pursuing their goals, and about how to target interventions effectively.

## Data availability statement

The datasets presented in this study can be found in online repositories. The names of the repository/repositories and accession number(s) can be found below: Materials and data for Study S1 are available at https://osf.io/jse96/?view_only=32ccbaa47aca463c8b4c844c46d71d70. Materials for Studies 1 and 2 and data for Study 1 are available at https://osf.io/x5ukf/?view_only=ce9a58ebd4af4daf8cbdd869ec41fec9. We are not able to publicly post the data for Study 2, although we can share data upon request and with IRB approval. The institutional IRB will not allow us to publicly post the data for this study due to the inclusion of course grades (FERPA considerations) and the language we used in our consent forms. We state in the paper that we can share the data for Study 2 upon request and with IRB approval.

## Ethics statement

The studies involving human participants were reviewed and approved by the University of Waterloo Research Ethics Board and The Ohio State University Institutional Review Board. The patients/participants provided their written informed consent to participate in this study.

## Author contributions

JR and TN conceptualized the research design, collected and analyzed the data, and drafted the initial version of the manuscript. KF, DM, and AS supervised this research, participating in developing the research design, interpreting of results, and writing revisions. ME aided with data analysis and interpretation. All authors contributed to the article and approved the submitted version.

## Funding

This work was supported by funding from the Social Sciences and Humanities Research Council of Canada Doctoral Fellowship Program (awarded to JR), the National Science Foundation Graduate Research Fellowship Program (awarded to TN), the Social Sciences and Humanities Research Council of Canada (awarded to AS; grant no. 435–2017-0184), the James S. McDonnell Foundation (awarded to DM; Collaborative grant no. 220020483), and the National Science Foundation (awarded to KF; grant no. 1626733).

## Conflict of interest

The authors declare that the research was conducted in the absence of any commercial or financial relationships that could be construed as a potential conflict of interest.

## Publisher’s note

All claims expressed in this article are solely those of the authors and do not necessarily represent those of their affiliated organizations, or those of the publisher, the editors and the reviewers. Any product that may be evaluated in this article, or claim that may be made by its manufacturer, is not guaranteed or endorsed by the publisher.

## Footnotes

^1^The promotion and prevention motivational systems are conceptualized as two independent systems, such that at a chronic level individuals can be high in one, relatively high in both, or relatively low in both (Higgins, 1997; Higgins et al., 2001). At any given moment, however, due to either chronic individual differences or situational factors, an individual is believed to be either in a promotion or prevention focused motivational state (Scholer et al., 2019).

^2^ https://osf.io/x5ukf/?view_only=ce9a58ebd4af4daf8cbdd869ec41fec9

^3^The attrition rates varied significantly between the two samples because of some unintended idiosyncrasies with the way the 2-part study was set-up in the participant pool for Study 1a. For Study 1a, many participants who completed Session were unable to sign-up for Session 2. 311 participants completed Session 1 of the study, but many of those participants had received their maximum allowance of credits and were not permitted to automatically sign up for Session 2. One of the authors worked with the participant pool program coordinator to implement a manual override to allow participants to sign-up for Session 2 by sending group and individual emails to the original participants. This effort resulted in 171 participants who completed both sessions for Study 1a (two of those participants did not complete any measures in Session 1, resulting in 169 total participants for Study 1a). In Study 1b, this issue was addressed (see Procedure section) and attrition was notably less; 167 of the 234 participants who completed Session 1 also completed Session 2. Participants who participated in Study 1a were unable to participate in Study 1b.

^4^This dataset has also been used in a paper examining the relation between construal level metamotivational knowledge and performance—citation removed for blinding (Nguyen et al., 2022).

^5^Some participants met more than one of the exclusion criteria.

^6^Given the unexpected retention of only 62% of our sample with the exclusion criteria outlined above, we conducted and reported analyses in the SOM that used only a subset of the exclusion criteria (i.e., those who did not consent to share grades with us, those whose academic records we could not retrieve, and those who did not report a high school GPA). We note that the patterns of data in the SOM are largely consistent with results in the main text, and if anything, are stronger given the increase in statistical power (retention of 88% of the sample; *N* = 520).
