# Corrigendum: The relationship between metamotivational knowledge and performance

**DOI:** 10.3389/fpsyg.2023.1271795

**Published:** 2023-08-16

**Authors:** Jessica Ross, Tina Nguyen, Kentaro Fujita, David B. Miele, Michael C. Edwards, Abigail A. Scholer

**Affiliations:** ^1^University of Waterloo, Waterloo, ON, Canada; ^2^The Ohio State University, Columbus, OH, United States; ^3^Boston College, Chestnut Hill, MA, United States; ^4^Arizona State University, Tempe, AZ, United States

**Keywords:** metamotivation, motivation, self-regulation, regulatory focus, performance

In the published article, there was an error in the author list. Author Abigail A. Scholer was erroneously placed as second from last author, instead of being placed as last author.

The authors apologize for this error and state that this does not change the scientific conclusions of the article in any way. The original article has been updated.

