# Supplementary material for: The relationship between metamotivational knowledge and performance
Source: Front Psychol. 2023 Jun 9;14:1124171. doi: 10.3389/fpsyg.2023.1124171 (PMC10289196; doi:10.3389/fpsyg.2023.1124171)
Supplement: Supplementary file 1 [file Data_Sheet_1.PDF]

The Relationship between Metamotivational Knowledge and Performance

# Supplemental Online Materials

## Table of Contents


Supplemental Study 1 Materials ........ 3
Regulatory Focus Knowledge Assessment ........ 3
Supplemental Study 1: Psychometric Evaluation of Metamotivational Knowledge Assessment .. 5
Method ........ 5
Participants ........ 5
Materials ........ 7
Results ........ 8
Exploratory and Confirmatory Factor Analyses ........ 8
Model Comparison and Measurement Invariance ........ 10
Analytic Approach ........ 10
Metamotivational Knowledge Assessment ........ 16
Test-Retest Reliability ........ 18
Discriminant Validity ........ 18
Study 1 Supplemental Materials ........ 21
Regulatory Focus Knowledge Assessment ........ 21
Additional Measures ........ 22
Study 1 Supplemental Analyses ........ 23
Sample Comparisons ........ 23
Is Time between Sessions related to Key Variables? ........ 23
Does Metamotivational Knowledge Differ for Participants Who Completed Both Sessions Versus Session 1 Only? ........ 25
Total Metamotivational Knowledge Predicting Overall Performance – Study Level Analysis 26
How does Total Metamotivational Knowledge Relate to Performance? ........ 27
Total Knowledge Predicting Brainstorming: Composite ........ 28
Total Knowledge Predicting Brainstorming: Number of Ideas ........ 29
Total Knowledge Predicting Brainstorming: Originality ........ 29
Total Knowledge Predicting Proofreading: Total Errors ........ 30
Total Knowledge Predicting Proofreading: Surface Errors ........ 31
Total Knowledge Predicting Proofreading: Contextual Errors ........ 32

How do Eager and Vigilant Metamotivational Knowledge Relate to Performance? ............... 32
Eager and Vigilant Knowledge Predicting Overall Performance: Study Level Analyses .... 32
Eager and Vigilant Knowledge Predicting Proofreading: Total Errors – Study Level Analysis ........................................................................................................................ 33
Eager and Vigilant Knowledge Predicting Proofreading: Surface Errors ............................ 34
Eager and Vigilant Knowledge Predicting Proofreading: Contextual Errors........................ 36
Study 2 Supplemental Materials ........................................................................................ 37
Regulatory Focus Knowledge Assessment .................................................................... 37
Additional Measures ........................................................................................................ 38
Study 2 Supplemental Analyses......................................................................................... 39
Using a Subset of the Exclusion Criteria from the Main Text ......................................... 39
Including Participants with Unusual Degrees of Missing Responses (> 50%)......................... 45
Additional Analyses Including Extent to Which Participants Took Study Seriously............... 50
Sample Reported in Main Text (N = 368)........................................................................ 50
Sample Reported in the SOM (N = 520) ........................................................................ 52
Pilot Study Materials (Follow up for Study 2 Presented in Study 2 Introduction)...................... 54
Pilot Study Results............................................................................................................ 56
References........................................................................................................................ 58

## Supplemental Study 1 Materials

### Regulatory Focus Knowledge Assessment

Instructions

In this part of the study, we will give you a description of a task. After that description, you will be presented with a preparatory activity or way of approaching the task. Then you will rate how useful the activity is for the goal of performing well on the task.

Structure

*Participants are presented with a task description, followed by all possible recall activities, and are asked to rate on a scale from 1 (extremely unhelpful) to 7 (extremely helpful) how useful each activity is for performing well on the task (note: only the end points of the scale have descriptors). They repeat this for each task description.*

*Task Description*:
Your goal is to be as accurate as possible by making sure to avoid lurking errors and pitfalls.

*Activity:*
Please write about a time in the past when you felt you made progress toward being successful in life.

*DV:* How useful is this activity for the goal of performing well on this task?
1 (extremely unhelpful) - 2 – 3 – 4 – 5 – 6 – 7 (extremely helpful)

*Eager Task Descriptions:*

1. Your goal is to be as creative as possible by seizing opportunities to take the ordinary and innovate.
2. Your goal is to imagine a future no one has seen before by seeing possibilities and occasions for advancement.
3. Imagine that you will complete a brainstorming task in which your goal is to be as creative as possible. Your goal is to be eager and open as you consider all possibilities!
4. Imagine that you work for an advertising firm and are responsible for pitching a new ad campaign to a major client. The client wants the campaign to be witty, innovative, and completely novel—unlike anything that's been seen on television before.

*Vigilant Task Descriptions:*

1. Your goal is to be as accurate as possible by making sure to avoid lurking errors and pitfalls.
2. Your goal is to be precise and make sure that you don't make a wrong turn in figuring out the right next step.
3. Imagine that you will complete a proofreading task in which your goal is to be as accurate as possible. Your goal is to be careful and thorough and be sure not to miss anything!
4. Imagine you are working in a plant that deals with volatile and potentially dangerous materials. Your job is to figure out the best system for managing workers who inspect products to ensure that they adhere to all safety standards.

*Promotion Recall Activities:*

1. Think about a time in the past when trying to achieve something important to you, you performed as well as you would like to do.
2. Think about a time in the past when compared to most people you were able to get what you wanted out of life.
3. Think about the hopes and aspirations that your parents or caregivers had for you growing up.

*Prevention Recall Activities:*

1. Think about a time in the past when you stopped yourself from acting in a way that your parents would have considered objectionable.
2. Think about a time when you were able to anticipate potential obstacles that led you to be more successful than you otherwise would have been.
3. Think about the duties and obligations your parents or caregivers expected you to uphold growing up.

## Supplemental Study 1: Psychometric Evaluation of Metamotivational Knowledge Assessment

We conducted a psychometric assessment of the regulatory focus task-motivation fit metamotivational knowledge measure using a sample of MTurk workers (participants from an internet-based crowdsourcing data acquisition platform; Litman et al., 2017) from a larger panel study. We split the sample into two subsamples to conduct confirmatory factor analyses (CFA) to determine which factor-structure best fits the data: a one-factor model, a two-factor model based on recall activity (i.e., a promotion factor and a prevention factor), a two-factor model based on task (i.e., an eager factor and a vigilant factor), or a four-factor model based on task and recall activity consistent with theoretical expectations (i.e., eager-prevention, eager-promotion, vigilant-prevention, vigilant-promotion). The confirmatory factor structure was also evaluated with the student samples from Studies 1 and 2 in the main text.

We also evaluated test-retest reliability of the assessment with a subset of the larger panel sample who completed the assessment one year apart. Finally, we evaluated the discriminant validity of the metamotivational knowledge assessment in a subset of the larger panel sample by examining its correlation with 33 personality and self-regulation measures (e.g., promotion and prevention focus, self-control, conscientiousness, proactive personality; see Table S2). Materials and data are available at https://osf.io/jse96/?view_only=32ccbaa47aca463c8b4c844c46d71d70.

### Method

#### *Participants*

The sample[1] consisted of 718 MTurk workers ($M_{age}$ = 42.93, $SD_{age}$ = 12.75; 397 women, 315 men, 5 non-binary people, 1 did not report; 79.2% White/European American, 5.8%

[1] This dataset has also been used in a paper examining the relation between construal level metamotivational knowledge and performance – citation removed for blinding.

Black/African American, 6.3% Asian American, 4.7% mixed racial/ethnic identity, 3.3% Hispanic/Latinx, 0.3% Native American; 0.3% did not report) who received payment for participating and consented to share their de-identified data. Additionally, subsets of Sample A completed paid follow-up sessions seven months later ($N$ = 528; $M_{age}$ = 43.76, $SD_{age}$ = 12.95; 285 women, 239 men, 3 non-binary people, 1 did not report; 80.5% White/European American, 5.1% Black/African American, 6.4% Asian American, 4.5% mixed racial/ethnic identity, 3.0% Hispanic/Latinx, 0.2% Native American; 0.2% did not report), nine months later ($N$ = 513; $M_{age}$ = 43.70, $SD_{age}$ = 12.79; 284 women, 225 men, 4 non-binary people; 78.2% White/European American, 6.0% Black/African American, 6.6% Asian American, 5.5% mixed racial/ethnic identity, 2.9% Hispanic/Latinx, 0.4% Native American; 0.4% did not report), and one year later ($N$ = 458; $M_{age}$ = 43.65, $SD_{age}$ = 12.70; 256 women, 199 men, 3 non-binary people; 77.7% White/European American, 7.0% Black/African American, 6.3% Asian American, 4.8% mixed racial/ethnic identity, 3.7% Hispanic/Latinx, 0.4% did not report).

**Exclusion Criteria.** Consistent with lab research practices for online studies for the lab that collected this data, we made *a priori* decisions to exclude a subset of participants across all analyses. Specifically, we planned to exclude participants who reported not paying attention during the study (i.e., reported being “very” or “extremely” distracted or taking the study “not at all” or “a little” seriously; $n$ = 0) and those who reported that they were not fluent in English ($n$ = 0). Additional exclusion criteria were applied given concerns about data quality on MTurk (Moss & Litman, 2020)—i.e., those with duplicate IP addresses ($n$ = 0), those who failed an English proficiency check ($n$ = 5), those who failed specific attention checks (i.e., “Please select strongly disagree”; $n$ = 0), and those who failed age consistency questions (i.e., reporting an age that is inconsistent with the birth year by > 1 year; $n$ = 0). As a result of these exclusion criteria, the

Panel sample had a final *N* = 713 ($M_{age}$ = 42.91, $SD_{age}$ = 12.75; 395 women, 312 men, 5 non-binary people, 1 did not report; 79.2% White/European American, 5.8% Black/African American, 6.3% Asian American, 4.8% mixed racial/ethnic identity, 3.4% Hispanic/Latinx, 0.3% Native American; 0.3% did not report). After attention-based exclusions, Sample A (7 months after baseline) had a final *N* = 521 ($M_{age}$ = 43.78, $SD_{age}$ = 12.94; 283 women, 234 men, 3 non-binary people, 1 did not report; 80.8% White/European American, 4.8% Black/African American, 6.3% Asian American, 4.6% mixed racial/ethnic identity, 3.1% Hispanic/Latinx, 0.2% Native American, 0.2% did not report), Sample A (9 months after baseline) had a final *N* = 506 ($M_{age}$ = 43.72, $SD_{age}$ = 12.80; 281 women, 221 men, 4 non-binary people; 78.6% White/European American, 5.9% Black/African American, 6.5% Asian American, 5.5% mixed racial/ethnic identity, 3.0% Hispanic/Latinx, 0.4% Native American, 0.4% did not report), and Sample A (1 year after baseline) had a final *N* = 449 ($M_{age}$ = 43.63, $SD_{age}$ = 12.74; 250 women, 196 men, 3 non-binary people; 78.0% White/European American, 6.7% Black/African American, 6.2% Asian American, 4.9% mixed racial/ethnic identity, 3.8% Hispanic/Latinx, 0.4% did not report).

### *Materials*

**Metamotivational Knowledge Assessment**. Participants completed a knowledge assessment of regulatory focus similar to Studies 1 and 2 in the main text, based on Scholer and Miele (2016). This assessment was completed at Time 1 (November 2019; final *N* = 713) and again at a one-year follow-up (Time 2, November 2020; final *N* = 449). Consistent with Study 2, participants rated the usefulness of recall activities for task performance (1 = *extremely unhelpful*, 7 = *extremely helpful*). This measure added one eager and one vigilant task to the existing tasks used in Study 2 and made minor revisions to the recall activities, such that the

assessment included eight tasks (4 eager, 4 vigilant) and six recall activities (3 promotion focus, 3 prevention focus), for a total of 48 randomly presented task and recall activity pairs.

**Additional Measures.** Participants also completed several measures of self-regulation, emotion regulation, and personality to examine their relationships with metamotivational knowledge in June 2020 (final $N$ = 521), August 2020 (final $N$ = 506), and October 2021 (final $N$ = 421). The full list of measures can be found in Table S2.

## Results

### *Confirmatory Factor Analyses*

To conduct initial CFAs in the Panel sample, we split the sample into two subsamples, ensuring that demographics and metamotivational knowledge did not differ across the two. We first parceled the items, which is a technique that involves using theory to aggregate items into composites to use as indicators (e.g., Little et al., 1999). The assessment in the Panel included 48 task-recall activity pairs (4 eager tasks, 4 vigilant tasks, 3 promotion recall activities, and 3 prevention recall activities). Based on theory, we parceled the assessment by averaging ratings for three recall activities for each task (e.g., using a single average rating of 3 promotion recall activities for an eager task rather than 3 ratings of promotion recall activities for an eager task). This resulted in 16 task-recall activity indicators: four indicators of the usefulness of promotion focus for eager tasks, four indicators of the usefulness of prevention focus for eager tasks, four indicators of the usefulness of promotion focus for vigilant tasks, and four indicators of the usefulness of prevention focus for vigilant tasks. This four-factor model (eager-prevention, eager-promotion, vigilant-prevention, vigilant-promotion) resulted in good fit in the first subset of the Panel (see Figure S1), $\chi^2(98) = 379.40$, $p < .001$, RMSEA = .09, SRMR = .04, CFI = .95.

We then tested the model using the other subset from the Panel. A four-factor model once again resulted in good fit, $\chi^2(98) = 273.85$, $p < .001$, RMSEA = .07, SRMR = .04, CFI = .96.

We also conducted a CFA using the student samples from Studies 1 and 2. Although the assessments differed in minor ways across the Panel and student samples[2], each assessment had the same overall structure and included items that were theoretically derived to measure participants' understanding of how to create regulatory focus task-motivation fit using tasks and manipulations from the empirical literature (e.g., Freitas & Higgins, 2002; Freitas et al., 2002; Higgins et al., 2001, 1994). Thus, for both student samples, we used the theory-consistent CFA model from the Panel sample. Similar to our treatment of the Panel data, we parceled the assessment in the student samples by averaging ratings for the recall activities for each task, resulting in 8 task-recall activity indicators in Study 1 and 12 task-recall activity indicators in Study 2. Given that there were only two indicators for each of the four factors in Study 1, the factor loadings within each factor were constrained to be equal. The four-factor model for both Studies 1 and 2 resulted in "acceptable" to "good" fit, suggesting some level of flexibility in the type of items that can be used to assess the latent constructs that reflect metamotivational knowledge (see Figure S1), *Study 1*: $\chi^2(14) = 147.65$, $p < .001$, RMSEA = .15, SRMR = .04, CFI = .92; *Study 2*: $\chi^2(48) = 206.86$, $p < .001$, RMSEA = .10, SRMR = .07, CFI = .92. We note that the RMSEA value for the model in Study 1 was above the cutoff for "acceptable" fit whereas the CFI was within the range for "acceptable" fit. That is, the RMSEA and CFI values disagreed and

[2] The assessment in Study 1 was the same as the original assessment used in Scholer and Miele (2016). The assessments used in Study 2 and with the S1 Panel sample were slightly modified to include more concrete and consequential tasks. Specifically, the assessment in Study 1 included 48 task-recall activity pairs based on the following number of tasks and recall activities: two eager tasks, two vigilant tasks, four promotion recall activities, four prevention recall activities, and four neutral/theoretically irrelevant recall activities (these neutral strategies are not modeled in the CFA because they are not used to calculate knowledge). The assessment in Study 2 also included 48 task-recall activity pairs based on the following: three eager tasks, three vigilant tasks, four promotion recall activities, and four prevention recall activities.

reflected qualitatively inconsistent interpretations of the fit of the model. One potential explanation could be the difference in the number of indicators per factor in Study 1 compared to Study 2 or the Panel sample. It is also possible that the RMSEA and CFI disagreed simply because these indexes evaluate model fit from different perspectives (for a more in-depth discussion of this issue, see Lai & Green, 2016). Given these possibilities, we compared the four-factor model to three other plausible models in the following section. To preview, across all samples, the four-factor model resulted in the best fit compared to all other models.

***Model Comparison and Measurement Invariance***

We compared the four-factor model to three other plausible models: a one-factor model, a two-factor model based on recall activity (i.e., a promotion factor and a prevention factor), and a two-factor model based on task (i.e., an eager factor and a vigilant factor). Across samples, the four-factor model was superior to all three alternative models, all $p$'s < .001 (see Models 1-16 in Table S1 for details). Additionally, taking demographics such as gender, race, and age into account, we assessed three types of measurement invariance: configural invariance (is model fit similar between groups?), metric invariance (does equating factor loadings between groups reduce model fit?), and scalar invariance (does equating factor intercepts between groups reduce model fit?). Generally, between-group analyses in the Panel sample revealed measurement invariance across gender, race, and age (see Models 1-15 in Table S2 for details). There was one exception with a scalar invariance test (Model 15): equating factor intercepts across age significantly decreased model fit, although not in a consequential manner (that is, model fit was still good across ages: CFI's > .90 and SRMR's < .08.

***Analytic Approach***

We recognize that this 4-factor model is just one way to assess the psychometric properties of this kind of measure of metamotivational knowledge in the regulatory focus domain. For transparency, after conducting the analyses presented above, we also explored alternative models that were not as robust as the model presented in this supplemental document. For instance, we wondered whether explicitly accounting for recall activity, task variance, or promotion/prevention focus might provide a better fitting model, so we tested additional models including: 6-factor models grouped by recall activities, 8-factor models grouped by tasks, and a bi-factor model comprised of a promotion factor and a prevention factor. With all of these models, there were issues with convergence and/or instability within or across samples. In other words, none of these models provided a viable or improved approach to modeling the data.

As Stadler et al. (2021) recently argued, the various aspects of one's knowledge in a particular domain may be acquired independently of each other and, therefore, may not cohere into a latent construct that has a causal effect on the indicators used to assess it. Instead, domain knowledge may be better characterized by a formative (rather than a reflective) measurement model. As Stadler et al. explain, "the causal direction implied by [such a] model is that the manifest observations form the latent construct. For example, teaching a student about genetics would increase his or her latent domain knowledge about biology but would not necessarily increase the student's knowledge about ecology" (p. 2). Similarly, when a student learns that a promotion focus is particularly adaptive for completing eager tasks, this does not necessarily mean that they also learn (at that particular point in time) that a prevention focus is less adaptive for completing such tasks, or that a prevention focus is particularly adaptive for completing vigilant tasks.

Although formative measurement can be controversial (see Bollen & Diamantopoulos, 2017) and we did not test any formative models of our metamotivational knowledge assessment, we believe that it offers a conceptual framework to explain why our alternate models did not exhibit better fit. Thus, for the purposes of the present studies, we retained the analytic approach used in past work to examine individual differences in metamotivational knowledge. This approach is based on the original analyses that were conducted by Scholer and Miele (2016) to establish that, on average, participants seem to understand the normative benefits of establishing regulatory focus task-motivation fit. The significant task type x motivation interaction that has consistently emerged in such analyses has been interpreted as evidence that participants possess normatively accurate metamotivational knowledge on average. To explore whether individual differences in this metamotivational knowledge are correlated with other variables, Scholer, Miele, and colleagues (e.g., Scholer & Miele, 2016; Jansen et al., 2022) have computed an overall metamotivational knowledge index based on the four composites ([eager knowledge: promotion recall preferences for eager tasks – prevention recall preference for eager tasks] + [vigilant knowledge: prevention recall preferences for vigilant tasks – promotion recall preferences for vigilant tasks]). We used this same formula to compute an index of metamotivational knowledge in the present paper.

Note that this index is equivalent to the interaction score for each participant, and thus allows us to capture the extent to which participants recognize the normative “if-then” contingencies in this context. The overall index succinctly captures the four factors from the CFA and reflects individual differences in normative knowledge of regulatory focus task-motivation fit. Thus, our primary analyses use this measure (for full transparency we also present analyses looking at eager knowledge and vigilant knowledge indices separately).

**Figure S1**

*Four-Factor Models of Metamotivational Knowledge of How to Create Regulatory Focus Task-Motivation Fit*

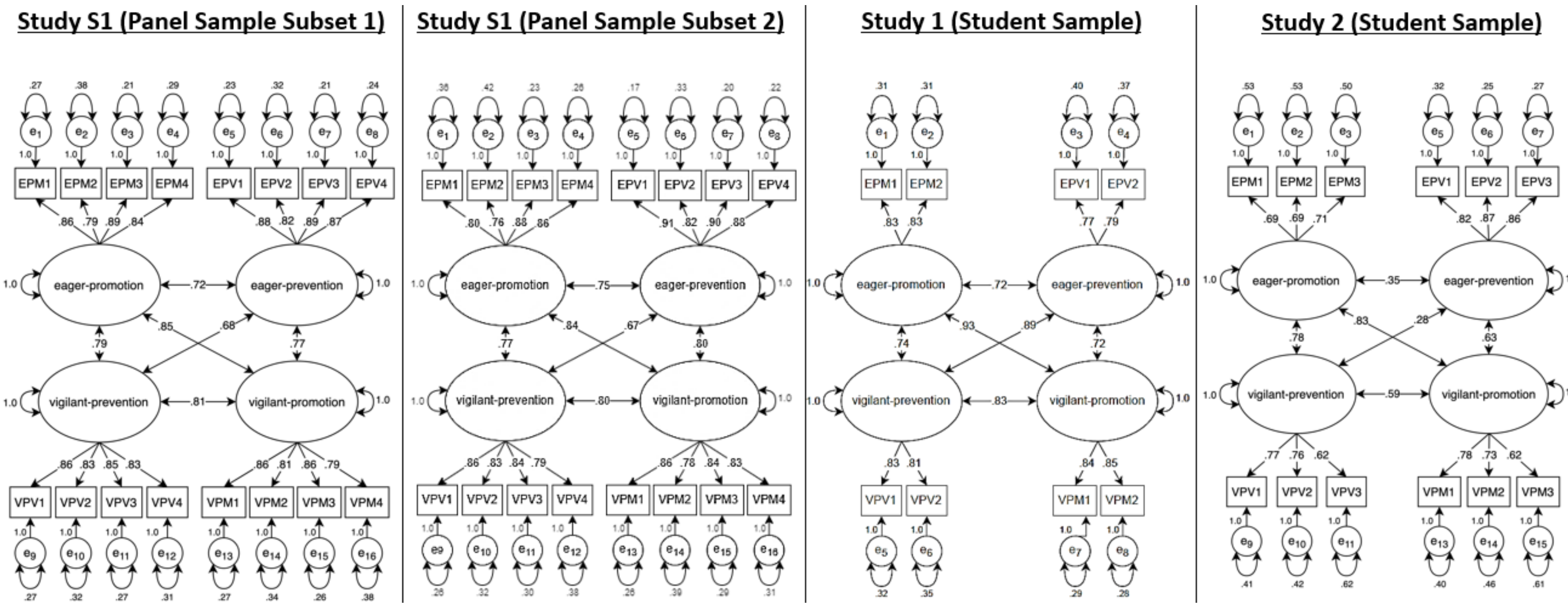


*Note.* (EPM = eager-promotion, EPV = eager-prevention, VPM = vigilant-promotion, VPV = vigilant-prevention). Figure S1 is identical to Figure 1 in the main text.

**Table S1**

*Confirmatory Factor Analyses Across Model Types (Panel and Student Samples)*

| Model | Description | $\chi^2$ | *df* | CFI | RMSEA | SRMR | Model Comparisons |
|---|---|---|---|---|---|---|---|
| Student Sample – Study 1 (*N* = 336) | | | | | | | |
| 1 | 1-factor model | 299.64 | 20 | 0.834 | 0.204 | 0.071 | Model 4 vs. 1: $\chi^2(2) = 151.99$, $p < .001$ |
| 2 | 2-factor model (by recall activity type) | 293.71 | 19 | 0.837 | 0.208 | 0.071 | Model 4 vs. 2: $\chi^2(1) = 31.06$, $p < .001$ |
| 3 | 2-factor model (by task type) | 178.72 | 19 | 0.905 | 0.158 | 0.046 | Model 4 vs. 3: $\chi^2(1) = 146.06$, $p < .001$ |
| **4** | **4-factor model (by task and recall activity)** | **147.65** | **18** | **0.923** | **0.147** | **0.041** | |
| Student Sample – Study 2 (*N* = 368) | | | | | | | |
| 5 | 1-factor model | 814.88 | 54 | 0.618 | 0.196 | 0.130 | Model 8 vs. 5: $\chi^2(6) = 608.02$, $p < .001$ |
| 6 | 2-factor model (by recall activity type) | 619.58 | 53 | 0.716 | 0.170 | 0.163 | Model 8 vs. 6: $\chi^2(5) = 412.71$, $p < .001$ |
| 7 | 2-factor model (by task type) | 747.64 | 53 | 0.651 | 0.189 | 0.173 | Model 8 vs. 7: $\chi^2(5) = 540.77$, $p < .001$ |
| **8** | **4-factor model (by task and recall activity)** | **206.86** | **48** | **0.920** | **0.095** | **0.068** | |
| Panel Sample (CFA subset #1: *N* = 357) | | | | | | | |
| 9 | 1-factor model | 1145.11 | 104 | 0.808 | 0.167 | 0.069 | Model 12 vs. 9: $\chi^2(6) = 765.71$, $p < .001$ |
| 10 | 2-factor model (by recall activity type) | 985.08 | 103 | 0.837 | 0.155 | 0.066 | Model 12 vs. 10: $\chi^2(5) = 605.67$, $p < .001$ |
| 11 | 2-factor model (by task type) | 971.64 | 103 | 0.840 | 0.154 | 0.064 | Model 12 vs. 11: $\chi^2(5) = 592.24$, $p < .001$ |
| **12** | **4-factor model (by task and recall activity)** | **379.40** | **98** | **0.948** | **0.090** | **0.038** | |
| Panel Sample (CFA subset #2: *N* = 356) | | | | | | | |
| 13 | 1-factor model | 1035.74 | 104 | 0.804 | 0.159 | 0.072 | Model 16 vs. 13: $\chi^2(6) = 761.89$, $p < .001$ |
| 14 | 2-factor model (by recall activity type) | 903.06 | 103 | 0.832 | 0.148 | 0.074 | Model 16 vs. 14: $\chi^2(5) = 577.42$, $p < .001$ |
| 15 | 2-factor model (by task type) | 851.28 | 103 | 0.843 | 0.143 | 0.069 | Model 16 vs. 15: $\chi^2(5) = 629.21$, $p < .001$ |
| **16** | **4-factor model (by task and recall activity)** | **273.85** | **98** | **0.963** | **0.071** | **0.036** | |

*Note.* CFI = Comparative Fit Index; RMSEA = Root Mean Square Error of Approximation; SRMR = Standardized Root Mean Square Residual. Generally, these models indicated acceptable to good fit: CFI > .90; RMSEA < .10; SRMR < .08 (Lai & Green, 2016). Table S1 is identical to Table 1 in the main text.

**Table S2**

*Measurement Invariance Tests for Gender, Race, and Age (Study S1: Panel Sample)*

| Model | Description | $\chi^2$ | *df* | CFI | RMSEA | SRMR | Model Comparisons |
|---|---|---|---|---|---|---|---|
| Gender | | | | | | | |
| 1 | Female participants ($n$ = 196) | 287.92 | 98 | 0.931 | 0.099 | 0.049 | |
| 2 | Male and other participants ($n$ = 161) | 231.01 | 98 | 0.951 | 0.092 | 0.035 | |
| 3 | Configural Invariance | 518.92 | 196 | 0.941 | 0.096 | 0.040 | |
| 4 | Metric Invariance | 531.74 | 208 | 0.941 | 0.093 | 0.049 | Model 3 vs. 4: $\chi^2(12) = 12.82$, $p = .382$ |
| 5 | Scalar Invariance | 541.13 | 220 | 0.941 | 0.090 | 0.049 | Model 4 vs. 5: $\chi^2(12) = 9.39$, $p = .669$ |
| Race | | | | | | | |
| 6 | White participants ($n$ = 283) | 298.95 | 98 | 0.954 | 0.085 | 0.036 | |
| 7 | All other participants ($n$ = 74) | 215.50 | 98 | 0.896 | 0.127 | 0.060 | |
| 8 | Configural Invariance | 514.46 | 196 | 0.942 | 0.095 | 0.039 | |
| 9 | Metric Invariance | 521.99 | 208 | 0.943 | 0.092 | 0.042 | Model 8 vs. 9: $\chi^2(12) = 7.53$, $p = .821$ |
| 10 | Scalar Invariance | 532.67 | 220 | 0.943 | 0.089 | 0.042 | Model 9 vs. 10: $\chi^2(12) = 10.68$, $p = .556$ |
| Age | | | | | | | |
| 11 | Participants < 41 years old ($n$ = 179) | 247.50 | 98 | 0.949 | 0.092 | 0.037 | |
| 12 | Participants > 40 years old ($n$ = 178) | 288.34 | 98 | 0.926 | 0.104 | 0.049 | |
| 13 | Configural Invariance | 535.84 | 196 | 0.938 | 0.099 | 0.040 | |
| 14 | Metric Invariance | 549.97 | 208 | 0.938 | 0.096 | 0.048 | Model 13 vs. 14: $\chi^2(12) = 14.14$, $p = .292$ |
| 15 | Scalar Invariance | 564.59 | 220 | 0.937 | 0.094 | 0.048 | Model 14 vs. 15: $\chi^2(12) = 14.61$, $p = .263$ |

*Note.* CFI = Comparative Fit Index; RMSEA = Root Mean Square Error of Approximation; SRMR = Standardized Root Mean Square Residual. Generally, these models indicated acceptable to good fit (CFI > .90, RMSEA < .08, SRMR < .08; Lai & Green, 2016)

***Metamotivational Knowledge Assessment***

**Metamotivational Knowledge of Regulatory Focus**. Consistent with prior work and with the student samples in the present research, the panel sample exhibited normatively accurate metamotivational knowledge of how to create regulatory focus task-motivation fit at both time points.

***Knowledge (Panel Time 1 – Nov 2019).*** To examine whether participants recognized how to create task-motivation fit, we submitted their usefulness ratings to a 2 (task: eager vs. vigilant) x 2 (recall activity: promotion vs. prevention) repeated measures ANOVA. Results revealed a main effect of task such that participants provided higher usefulness ratings for vigilant (*M*=4.30, *SD*=1.04) compared to eager tasks (*M*=4.14, *SD*=1.00), $F(1, 712)=47.12$, $p<.001$, $\eta_p^2=.06$. There was also a significant main effect of recall activity such that participants gave higher usefulness ratings for promotion (*M*=4.38, *SD*=1.00) compared to prevention recall activities (*M*=4.06, *SD*=1.03), $F(1, 712)=240.50$, $p<.001$, $\eta_p^2=.25$. As expected, results revealed a significant interaction between task and recall activity, $F(1, 712)=586.15$, $p<.001$, $\eta_p^2=.45$.

We first examined this interaction as a function of recall activity. As predicted, participants rated the promotion recall activities as more useful for eager tasks (*M*=4.58, *SD*=1.04) than for vigilant tasks (*M*=4.18, *SD*=1.08), $t(712)=15.20$, $p<.001$, $d=.57$. Participants also rated the prevention recall activities as more useful for vigilant tasks (*M*=4.41, *SD*=1.14) than for eager tasks (*M*=3.71, *SD*=1.15), $t(712)=19.32$, $p<.001$, $d=.72$. Next, we examined this interaction as a function of task. As expected, within eager tasks, participants rated promotion recall activities as more useful than prevention recall activities, $t(712)=27.07$, $p<.001$, $d=1.02$. By contrast, within vigilant tasks, participants rated prevention recall activities as more useful than promotion recall activities, $t(712)=7.72$, $p<.001$, $d=.29$. In sum, consistent with the student

samples presented in the main text, participants on average recognized how to create regulatory focus task-motivation fit.

***Knowledge (Panel Time 2 – Nov 2020).*** Similarly, for the one-year follow-up of the Panel sample, we submitted participants' usefulness ratings to a 2 (task: eager vs. vigilant) x 2 (recall activity: promotion vs. prevention) repeated measures ANOVA. All effects were consistent with Time 1 results. There was a significant main effect of task such that participants provided higher usefulness ratings for vigilant tasks (*M*=4.33, *SD*=.95) than for eager tasks (*M*=4.13, *SD*=.90), *F*(1, 448)=54.73, *p*<.001, $\eta_p^2$=.11. Results also revealed a main effect of recall activity such that participants gave higher usefulness ratings for promotion recall activities (*M*=4.43, *SD*=.90) than for prevention recall activities (*M*=4.03, *SD*=.95), *F*(1, 448)=208.61, *p*<.001, $\eta_p^2$=.32. Replicating results from other samples, there was a significant task x recall activity interaction, *F*(1, 448)=416.84, *p*<.001, $\eta_p^2$=.48.

We first examined this interaction as a function of recall activity. As predicted, participants rated the promotion recall activities as more useful for eager tasks (*M*=4.62, *SD*=.96) than for vigilant tasks (*M*=4.23, *SD*=.97), *t*(448)=11.17, *p*<.001, *d*=.53. Also consistent with expectations, participants rated the prevention recall activities as more useful for vigilant tasks (*M*=4.43, *SD*=1.07) than for eager tasks (*M*=3.63, *SD*=1.06), *t*(448)=17.64, *p*<.001, *d*=.83. Next, we examined this interaction as a function of task. As predicted, within eager tasks, participants rated promotion recall activities as more useful than prevention recall activities, *t*(448)=22.95, *p*<.001, *d*=1.08. By contrast, within vigilant tasks, participants rated prevention recall activities as more useful than promotion recall activities, *t*(448)=5.36, *p*<.001, *d*=.26. These findings are consistent with the Time 1 results from the Panel as well as the student samples reported in the main text.

### ***Test-Retest Reliability***

To examine the stability of regulatory focus task-motivation fit metamotivational knowledge, we examined the Pearson correlation coefficient between knowledge at baseline (Time 1, November 2019) and at the one-year follow-up (Time 2, November 2020; $N = 449$) in the Panel sample. Test-retest reliability of the knowledge assessment was .48 for total knowledge, .51 for eager knowledge, and .34 for vigilant knowledge, $p$'s < .001. These correlations reflect both stability and variability in metamotivational knowledge, as would be expected for beliefs that may change over time as a function of experience. Indeed, these correlations fall in the range one would expect for constructs expected to be characterized by both stability and lability. For instance, Kashdan et al. (2020) examined the temporal stability of the Personalized Psychological Flexibility Index (PPFI) by computing the Pearson correlation coefficient between each of the five-item PPFI subscales as well as the total score from baseline (Time 1) to 4- and 6-month follow-ups (Time 2) and found test-retest reliability in the range of .44 to .61. Thus, this assessment of knowledge indicated test-retest reliability over a year in the range that one would expect based on similar constructs that are influenced, in part, by time and experience.

### ***Discriminant Validity***

Recall that we also administered several measures of self-regulation, emotion regulation, and personality to examine their relationships with metamotivational knowledge. Table S3 displays the zero-order correlations between these measures and normative knowledge. All correlations with knowledge were relatively small, $r$'s < .15, demonstrating strong discriminant validity. That is, metamotivational knowledge appears to be a novel construct cannot be reduced to existing measures of self-regulation, emotion regulation, or personality.

**Table S3**

*Zero-Order Correlations (Panel Sample Only)*

| Scale | *N* | Details: Example item (scale points) | Total Knowledge | Eager Knowledge | Vigilant Knowledge |
|---|---|---|---|---|---|
| Self-Regulation Ability (Fishbach et al., 2003) | 521 | "To what extent are you successful at achieving your goals?" (1 = not at all successful, 7= extremely successful) | **-.10*** | -.06 | -.09 |
| Self-Control (Tangney et al., 2004) | 521 | "I am good at resisting temptation." (1 = does not describe me, 5 = describes me extremely well) | -.03 | -.05 | -.0003 |
| Lay Theories of Intelligence (Dweck & Leggett, 1988) | 521 | "You have a certain amount of intelligence, and you can't really do much to change it." (reverse-scored; 1 = strongly agree, 7 = strongly disagree) | .05 | .03 | .04 |
| Promotion Focus (Higgins et al., 2001) | 421 | "I feel like I have made progress toward being successful in my life." (1 = certainly false, 5 = certainly true) | .04 | **.11*** | -.06 |
| Prevention Focus (Higgins et al., 2001) | 421 | "How often did you obey rules and regulations that were established by your parents?" (1 = never or seldom, 5 = always) | .003 | .03 | -.03 |
| Mastery Approach Motivation (Elliot & Murayama, 2008) | 421 | "My goal is to learn as much as possible." (1 = completely disagree, 5 = completely agree) | .03 | .09 | -.05 |
| Mastery Avoidance Motivation (Elliot & Murayama, 2008) | 421 | "My aim is to avoid learning less than I possibly could." (1 = completely disagree, 5 = completely agree) | -.01 | .01 | -.03 |
| Performance Approach Motivation (Elliot & Murayama, 2008) | 421 | "My aim is to perform well relative to others." (1 = completely disagree, 5 = completely agree) | -.03 | -.03 | -.02 |
| Performance Avoidance Motivation (Elliot & Murayama, 2008) | 421 | "My aim is to avoid doing worse than others." (1 = completely disagree, 5 = completely agree) | -.04 | -.04 | -.01 |
| Behavioural Activation System (Carver & White, 2013) | 421 | "I go out of my way to get things I want." (1 = very true for me, 4 = very false for me) | -.05 | .01 | -.08 |
| Behavioural Inhibition System (Carver & White, 2013) | 421 | "I feel worried when I think I have done poorly at something important." (1 = very true for me, 4 = very false for me) | **.14**** | **.13**** | .06 |
| Behavioural Activation System (Corr & Cooper, 2015) | 421 | "I am often preoccupied with unpleasant thoughts." (1 = not at all, 4 = highly) | -.03 | .02 | -.08 |
| Behavioural Inhibition System (Corr & Cooper, 2015) | 421 | "I'm motivated to be successful in my personal life." (1 = not at all, 4 = highly) | .05 | .05 | .03 |
| Fight-Flight-Freeze System (Corr & Cooper, 2015) | 421 | "I am the sort of person who easily freezes-up when scared." (1 = not at all, 4 = highly) | -.02 | .05 | -.08 |
| Mindfulness (Brown & Ryan, 2003) | 421 | "I find it difficult to stay focused on what's happening in the present." (reverse-scored; 1 = almost always, 6 = almost never) | .01 | .03 | -.02 |
| Conscientiousness (MacCann et al., 2009) | 421 | "I demand quality." (1 = not at all like me, 5 = very much like me) | -.03 | -.02 | -.03 |
| Conscientiousness (Donnellan et al., 2006) | 506 | "I like order." (1 = very inaccurate, 5 = very accurate) | -.02 | -.01 | -.02 |
| Openness (Donnellan et al., 2006) | 506 | "I am not interested in abstract ideas." (reverse-scored; 1 = very inaccurate, 5 = very accurate) | .08 | **.09*** | .03 |
| Agreeableness (Donnellan et al., 2006) | 506 | "I sympathize with others' feelings." (1 = very inaccurate, 5 = very accurate) | **.09*** | **.11*** | .01 |
| Extraversion (Donnellan et al., 2006) | 506 | "I am the life of the party." (1 = very inaccurate, 5 = very accurate) | **-.12**** | -.06 | **-.12**** |

| | | | | | |
|---|---|---|---|---|---|
| Neuroticism (Donnellan et al., 2006) | 506 | "I have frequent mood swings." (1 = very inaccurate, 5 = very accurate) | .01 | .04 | -.03 |
| Proactive Personality (Seibert et al., 1999) | 506 | "I am constantly on the lookout for new ways to improve my life." (1 = strongly disagree, 7 = strongly agree) | -.08 | -.03 | **-.10*** |
| Hope (Snyder et al., 1991) | 521 | "I can think of many ways to get out of a jam." (1 = definitely false, 4 = definitely true) | **-.10*** | -.03 | **-.12**** |
| Grit - Perseverance (Duckworth & Quinn, 2009) | 521 | "I finish whatever I begin." (1 = does not describe me, 5 = describes me extremely well) | **-.10*** | -.06 | **-.09*** |
| Grit - Consistency (Duckworth & Quinn, 2009) | 521 | "I often set a goal but later choose to pursue a different one." (reverse-scored; 1 = does not describe me, 5 = describes me extremely well) | -.04 | -.02 | -.03 |
| John Henryism (James et al., 1983) | 521 | "I believe that hard work is the best possible way for a person to get ahead in life" (1 = does not describe me, 5 = describes me extremely well) | -.07 | -.02 | **-.09*** |
| Spontaneous Self-Distancing (Ayduk & Kross, 2010) | 521 | Recall rejection experience (1 = mainly immersed participant, 7 = mainly distanced observer) | -.03 | **-.13**** | **.10*** |
| Emotion Reappraisal (Gross & John, 2003) | 521 | "I control my emotions by changing the way I think about the situation I'm in." (1 = strongly disagree, 7 = strongly agree) | -.01 | -.02 | -.004 |
| Emotion Suppression (Gross & John, 2003) | 521 | "I keep my emotions to myself." (1 = strongly disagree, 7 = strongly agree) | -.08 | **-.09*** | -.03 |
| Positive Emotion Expression (Burton & Bonanno, 2016) | 521 | "A coworker gets a promotion and wants to talk about it." (1 = unable [to be even more expressive], 7 = very able [to be even more expressive]) | .08 | **.10*** | .02 |
| Negative Emotion Expression (Burton & Bonanno, 2016) | 521 | "You're attending the funeral of someone you don't know." (1 = unable [to be even more expressive], 7 = very able [to be even more expressive]) | .004 | .01 | -.01 |
| Positive Emotion Suppression (Burton & Bonanno, 2016) | 521 | "During a meeting with a supervisor, his/her phone unexpectedly begins to play an embarrassing ringtone." (1 = unable [to conceal], 7 = very able [to conceal]) | .03 | .03 | .02 |
| Negative Emotion Suppression (Burton & Bonanno, 2016) | 521 | "You are on a first date at a restaurant having dinner, and a stranger spills their drink on you." (1 = unable [to conceal], 7 = very able [to conceal]) | -.04 | -.05 | -.01 |

*Note:* *$p < .05$, **$p < .01$

## Study 1 Supplemental Materials

### Regulatory Focus Knowledge Assessment

Participants completed an assessment of their metamotivational knowledge of regulatory focus used in prior work (Scholer & Miele, 2016). Participants were told that they would see descriptions of tasks paired with a recall activity. For each pair, participants rated how much they would prefer to complete that recall activity (e.g., Please write about a time in the past when you felt you made progress toward being successful in life) before doing the task (e.g., Your goal is to be as creative as possible by seizing opportunities to take the ordinary and innovate) on a scale from 1 (not at all) to 7 (very much). The regulatory focus knowledge assessment consisted of four tasks (2 eager, 2 vigilant) and twelve recall activities (4 promotion focus, 4 prevention focus, 4 neutral). Thus, participants saw a total of 48 randomly presented task and recall activity pairs.

Eager Task Descriptions:

1. Your goal is to be as creative as possible by seizing opportunities to take the ordinary and innovate.
2. Your goal is to imagine a future no one has seen before by seeing possibilities and occasions for advancement.

Vigilant Task Descriptions:

1. Your goal is to be as accurate as possible by making sure to avoid lurking errors and pitfalls.
2. Your goal is to be precise and make sure that you don’t make a wrong turn in figuring out the right next step.

Promotion Recall Activities:

1. Please write about a time in the past when you felt you made progress toward being successful in life.
2. Please write about a time in the past when compared to most people you were able to get what you wanted out of life.
3. Please write about a time in the past when trying to achieve something important to you, you performed as well as you ideally would have liked to.
4. Please write about your hopes and aspirations as a child. What accomplishments did you ideally want to meet when you were a child?

<u>Prevention Recall Activities:</u>

1. Please write about a time in the past when being careful enough avoided getting you into trouble.
2. Please write about a time in the past when you stopped yourself from acting in a way that your parents would have considered objectionable.
3. Please write about a time in the past when you were careful not to get on your parents' nerves.
4. Please write about your duties and obligations as a child. What responsibilities did you think you ought to meet when you were a child?

<u>Neutral Recall Activities:</u>

1. Please describe what your kitchen looked like when you were a child.
2. Please describe the physical layout of the most recent restaurant you visited.
3. Please describe the various floor surfaces in your home.
4. Please describe the inside of the last bus on which you traveled.

## Additional Measures

Studies 1a and 1b contained additional exploratory measures beyond the primary construct of interest reported in the main text, specifically measures of lay beliefs of motivation (King, 2019) and proactive personality (Bateman & Crant, 1993).

## Study 1 Supplemental Analyses

### Sample Comparisons

Results revealed a consistent pattern such that the relation between metamotivational knowledge and performance was observed in Study 1a, but not Study 1b. There were no clear differences between the samples in terms of demographics or performance level that can easily explain this unpredicted difference (see Table S4).

**Table S4**

*Study-Level Descriptive Statistics*

| | Mean (SD) | | | |
|---|---|---|---|---|
| | Study 1a | Study 1b | *t* | *p* |
| Age | 20.14 (4.25) | 20.16 (4.23) | 0.04 | .966 |
| Proofreading Performance | 12.85 (6.44) | 11.94 (6.22) | 0.93 | .351 |
| Brainstorming Performance (Composite) | 5.68 (2.10) | 5.19 (2.37) | 1.40 | .163 |
| Brainstorming Performance (Number of Ideas) | 8.48 (4.20) | 7.26 (4.51) | 1.80 | .074 |
| Brainstorming Performance (Originality) | 3.00 (0.53) | 3.21 (0.73) | 1.15 | .252 |
| Metamotivational Knowledge | 0.79 (1.39) | 0.59 (1.29) | 1.40 | .164 |

### Is Time between Sessions related to Key Variables?

Because of idiosyncrasies related to the implementation of the two-part study in Study 1a, the time between Part 1 and Part 2 ranged from a few minutes to several weeks. This was controlled for in Study 1b, with all participants receiving a link to complete Session 2 three days after completing Session 1, with instructions to finish within seven days of receiving the e-mail. Due to the range of time between sessions both within and between studies, we examined whether time between sessions affected the results. Results for the full sample as well as each study separately showed that time was not significantly correlated with performance (see Table S5) and did not significantly interact with task type or knowledge to predict performance (see Table S6).

**Table S5**

*Zero-Order Correlations: Time Between Sessions and Performance*

| | Full Sample | Study 1a | Study 1b |
|---|---|---|---|
| Overall Performance | .05 | .08 | -.08 |
| | .392 | .295 | .281 |
| Brainstorming Performance | .05 | .07 | -.22 |
| (Composite) | .567 | .540 | .053 |
| Brainstorming Performance | .06 | .07 | -.20 |
| (Number of Ideas) | .477 | .504 | .071 |
| Brainstorming Performance | -.07 | -.04 | -.15 |
| (Originality) | .402 | .747 | .175 |
| Proofreading Performance | .07 | .09 | -.04 |
| | .391 | .402 | .728 |

**Table S6**

*Regression Analyses for Studies 1a and 1b: Metamotivational Knowledge Predicting Task Performance While Controlling for Task Type and Time Between Sessions*

| Study | Predictors | *b* | *SE* | β | *t* | *p* | 95% CI |
|---|---|---|---|---|---|---|---|
| 1a | Intercept | -0.04 | 0.09 | | -0.40 | .691 | [-0.21, 0.14] |
| | Task Type | -0.01 | 0.09 | -.01 | -0.14 | .886 | [-0.19, 0.17] |
| | Time | 0.0002 | 0.0002 | .09 | 1.21 | .227 | [-0.0001, 0.001] |
| | Total Knowledge | 0.16 | 0.06 | .23 | 2.60 | .010 | [0.04, 0.28] |
| | Knowledge*Time | -0.000003 | 0.0001 | -.02 | -0.24 | .809 | [-0.0002, 0.0002] |
| | Task Type*Time | -0.000003 | 0.0002 | -.002 | -0.02 | .984 | [-0.0003, 0.0003] |
| 1b | Intercept | 0.17 | 0.15 | | 1.15 | .254 | [-0.12, 0.45] |
| | Task Type | -0.13 | 0.14 | -.12 | -0.90 | .371 | [-0.40, 0.15] |
| | Time | -0.002 | 0.001 | -.20 | -1.85 | .066 | [-0.003, 0.0001] |
| | Total Knowledge | 0.09 | 0.10 | .12 | 0.90 | .369 | [-0.11, 0.30] |
| | Knowledge*Time | -0.0001 | 0.001 | -.03 | -0.24 | .815 | [-0.001, 0.001] |
| | Task Type*Time | 0.001 | 0.001 | .26 | 1.67 | .096 | [-0.0002, 0.0003] |

**Does Metamotivational Knowledge Differ for Participants Who Completed Both Sessions Versus Session 1 Only?**

The first analysis presented in Study 1 examines metamotivational knowledge of regulatory focus task-motivation fit. In the main text, we conducted this analysis on participants who completed both Parts 1 and 2. We also conducted these analyses for the full sample who completed Part 1 ($N$ = 558)—reported below; the pattern of results is the same.

***Full Sample***

To examine participants' metamotivational knowledge about regulatory focus, we submitted their preference ratings to a 2 (task: eagerness vs. vigilance) x 3 (recall activity: promotion vs. prevention vs. neutral) repeated measures ANOVA. Results revealed a main effect of recall type, $F(1.55, 829.52) = 64.80$, $p < .001$, $\eta_p^2 = .11$, revealing that participants preferred promotion activities ($M$ = 4.33, $SD$ = 1.30) to both prevention activities ($M$ = 3.78, $SD$ = 1.43) and neutral activities ($M$ = 3.71, $SD$ = 1.43) at the $p < .001$ level; preference for prevention and neutral activities did not significantly differ ($p = .286$). There was no main effect of task type, $F(1, 536) = 1.34$, $p = .247$, $\eta_p^2 = .002$. As predicted, results revealed a significant task x recall activity interaction, $F(1.81, 969.06) = 44.97$, $p < .001$, $\eta_p^2 = .08$.

Participants preferred promotion recall activities when anticipating an eager task ($M$ = 4.46, $SD$ = 1.39) relative to a vigilance task ($M$ = 4.19, $SD$ = 1.39), $t(536) = 6.56$, $p < .001$, $d$ = 0.28. In contrast, participants preferred prevention recall activities when anticipating a vigilance task ($M$ = 3.95, $SD$ = 1.43) relative to an eager task ($M$ = 3.58, $SD$ = 1.41), $t(536) = 7.79$, $p < .001$, $d$ = 0.34. There was no difference in preference for neutral recall activities when

anticipating vigilance tasks (*M* = 3.70, *SD* = 1.61) vs. eagerness tasks (*M* = 3.70, *SD* = 1.54), *t*(536) = 0.16, *p* = .871, *d* = 0.01.

Next, we tested simple effects as a function of task. Comparing promotion, prevention, and neutral recall activities for eager tasks, participants preferred promotion activities (*M* = 4.46, *SD* = 1.38) to both prevention activities (*M* = 3.59, *SD* = 1.41), *t*(538) = 16.27, *p* < .001, *d* = 0.70 , and neutral activities (*M* = 3.71, *SD* = 1.53), *t*(538) = 9.68, *p* < .001, *d* = 0.42; prevention and neutral ratings did not significantly differ, *t*(539) = 1.84, *p* = .066, *d* = 0.08. For vigilance tasks, participants once again preferred promotion activities (*M* = 4.19, *SD* = 1.39) to both prevention activities (*M* = 3.96, *SD* = 1.43), *t*(339) = 4.98, *p* < .001, *d* = 0.21, and neutral activities (*M* = 3.70, *SD* = 1.61), *t*(339) = 6.02, *p* < .001, *d* = 0.26. They also preferred prevention activities to neutral activities, *t*(339) = 3.27, *p* = .001, *d* = 0.14.

**Total Metamotivational Knowledge Predicting Overall Performance – Study Level Analysis**

As reported in the main text, we regressed participants' performance scores on study, task type, task skill, task enjoyment, task familiarity, total knowledge, and the interactions between total knowledge and both task type and study. As indicated in Table 3 in the main text, there was a marginal interaction between knowledge and study, indicating that the effect of knowledge on performance was likely moderated by study. Conducting the regression analyses separately for each study, knowledge emerged as a significant predictor of task performance in Study 1a (*b* = 0.19, *p* = .001), but not in Study 1b (*b* = 0.03, *p* = .599; see Table S7).

**Table S7**

*Regression Analyses for Studies 1a and 1b: Metamotivational Knowledge Predicting Task Performance While Controlling for Task Type, Skill, Enjoyment, and Familiarity*

| | Predictors | *b* | *SE* | β | *t* | *p* | 95% CI |
|---|---|---|---|---|---|---|---|
| Study 1a | Intercept | -0.26 | 0.18 | | -1.40 | .164 | [-0.62, 0.11] |
| | Total Knowledge | 0.19 | 0.06 | .27 | 3.41 | .001 | [0.08, 0.30] |
| | Task Type | 0.03 | 0.08 | .03 | 0.35 | .724 | [-0.13, 0.17] |
| | Task Skill | 0.06 | 0.08 | .07 | 0.74 | .458 | [-0.10, 0.23] |
| | Task Enjoyment | 0.09 | 0.06 | .13 | 1.47 | .144 | [-0.03, 0.21] |
| | Task Familiarity | 0.05 | 0.07 | .07 | 0.76 | .450 | [-0.08, 0.19] |
| | Knowledge*Task Type | 0.06 | 0.06 | .09 | 1.10 | .274 | [-0.05, 0.17] |
| Study 1b | Intercept | -.16 | .18 | | -0.90 | .370 | [-0.52, 0.20] |
| | Total Knowledge | .03 | .06 | .04 | 0.53 | .599 | [-0.01, 0.15] |
| | Task Type | -.01 | .08 | -.01 | -0.09 | .927 | [-0.16, 0.14] |
| | Task Skill | .18 | .09 | .20 | 2.02 | .045 | [0.004, 0.35] |
| | Task Enjoyment | .07 | .06 | .11 | 1.18 | .239 | [-0.05, 0.19] |
| | Task Familiarity | .03 | .06 | .04 | 0.43 | .670 | [0.10, 0.16] |
| | Knowledge*Task Type | .04 | .06 | .06 | 0.70 | .485 | [-0.08, 0.16] |

**How does Total Metamotivational Knowledge Relate to Performance?**

The main text presents the analyses for the relation between total knowledge and task performance, which was standardized using the composite score for the brainstorming task and total number of errors for the proofreading task. Here we present several additional analyses for full transparency. As can be seen in Tables S8, S9, and S10, there was no effect of total knowledge on any of the three brainstorming performance metrics (i.e., composite score, number of ideas, and originality), nor was there an interaction between knowledge and study. Total knowledge was a significant predictor of the total number of proofreading errors detected (see

Table S11, and these results do not differ as a function of proofreading performance metric, i.e., surface vs. complex errors; see Tables S12 and S13). There was a marginal interaction between total knowledge and study for both the total number of proofreading errors and number of surface-level errors. Results revealed a pattern similar to that of the analyses in the main text, such that there was an effect of knowledge in Study 1a but not 1b.

### ***Total Knowledge Predicting Brainstorming: Composite***

**Table S8**

*Regression Analyses Predicting Brainstorming Performance (Composite Score) from Total Knowledge, Controlling for Study and Task Skill, Enjoyment, and Familiarity*

| Predictors | *B* | *SE* | β | *t* | *p* | 95% CI |
|---|---|---|---|---|---|---|
| Intercept | 5.61 | 0.45 | | 12.37 | <.001 | [4.71, 6.50] |
| Total Knowledge | 0.18 | 0.14 | .11 | 1.30 | .195 | [-0.09, 0.45] |
| Study | -0.24 | 0.17 | -.11 | -1.38 | .170 | [-0.58, 0.10] |
| Task Skill | 0.01 | 0.21 | .002 | 0.03 | .980 | [-0.41, 0.42] |
| Task Enjoyment | -0.07 | 0.15 | -.04 | -0.46 | .646 | [-0.37, 0.23] |
| Task Familiarity | 0.47 | 0.18 | .26 | 2.68 | .008 | [0.12, 0.82] |
| Knowledge*Study | -0.17 | 0.13 | -.10 | -1.27 | .205 | [-0.44, 0.09] |

***Total Knowledge Predicting Brainstorming: Number of Ideas***

**Table S9**

*Regression Analyses Predicting Brainstorming Performance (Number of Ideas) from Total Knowledge, Controlling for Study and Task Skill, Enjoyment, and Familiarity*

| Predictors | *B* | *SE* | β | *t* | *p* | 95% CI |
|---|---|---|---|---|---|---|
| Intercept | 8.27 | 0.87 | | 9.52 | <.001 | [6.56, 10.0] |
| Total Knowledge | 0.35 | 0.26 | .11 | 1.35 | .179 | [-0.16, 0.87] |
| Study | -0.52 | 0.33 | -.12 | -1.57 | .118 | [-1.18, 0.13] |
| Task Skill | 0.10 | 0.40 | .03 | 0.25 | .801 | [-0.69, 0.89] |
| Task Enjoyment | -0.19 | 0.29 | -.06 | -0.64 | .523 | [-0.76, 0.39] |
| Task Familiarity | 0.83 | 0.34 | .24 | 2.44 | .016 | [0.16, 1.49] |
| Knowledge*Study | -0.32 | 0.26 | -.10 | -1.23 | .220 | [-0.82, 0.19] |

***Total Knowledge Predicting Brainstorming: Originality***

**Table S10**

*Regression analyses predicting brainstorming performance (originality) from total knowledge, controlling for study and task skill, enjoyment, and familiarity*

| Predictors | *B* | *SE* | β | *t* | *p* | 95% CI |
|---|---|---|---|---|---|---|
| Intercept | 2.98 | 0.13 | | 22.57 | <.001 | [2.72, 3.24] |
| Total Knowledge | -0.01 | 0.04 | -.02 | -0.17 | .864 | [-0.09, 0.07] |
| Study | 0.06 | 0.05 | .09 | 1.15 | .251 | [-0.04, 0.16] |
| Task Skill | -0.03 | 0.06 | -.05 | -0.51 | .613 | [-0.15, 0.09] |
| Task Enjoyment | 0.03 | 0.04 | .06 | 0.64 | .522 | [-0.06, 0.12] |
| Task Familiarity | 0.09 | 0.05 | .18 | 1.85 | .066 | [-0.01, 1.00] |
| Knowledge*Study | -0.01 | 0.04 | -.02 | -0.28 | .784 | [-0.09, 0.07] |

***Total Knowledge Predicting Proofreading: Total Errors***

**Table S11**

*Regression Analyses Predicting Proofreading Performance from Total Knowledge, Controlling for Study and Task Skill, Enjoyment, and Familiarity*

| | Predictors | *B* | *SE* | β | *t* | *p* | 95% CI |
|---|---|---|---|---|---|---|---|
| Full Sample | Intercept | 9.69 | 1.01 | | 9.64 | <.001 | [7.71, 11.68] |
| | Total Knowledge | 0.98 | 0.34 | .21 | 2.86 | .005 | [0.30, 1.66] |
| | Study | -0.41 | 0.44 | -.07 | -0.93 | .357 | [-1.28, 0.47] |
| | Task Skill | 1.10 | 0.49 | .20 | 2.26 | .025 | [0.14, 2.06] |
| | Task Enjoyment | 1.01 | 0.34 | .26 | 2.96 | .003 | [0.34, 1.67] |
| | Task Familiarity | -0.29 | 0.35 | -.06 | -0.81 | .417 | [-0.98, 0.41] |
| | Knowledge*Study | -0.65 | 0.34 | -.14 | -1.90 | .059 | [-1.33, 0.03] |
| Study 1a | Intercept | 9.77 | 1.43 | | 6.81 | <.001 | [6.91, 12.62] |
| | Total Knowledge | 0.71 | 0.68 | .13 | 1.04 | .301 | [-0.65, 2.06] |
| | Task Skill | 1.16 | 0.48 | .29 | 2.41 | .018 | [0.20, 2.13] |
| | Task Enjoyment | -0.37 | 0.53 | -.08 | -0.70 | .489 | [-1.42, 0.69] |
| | Task Familiarity | 1.65 | 0.55 | .31 | 3.03 | .003 | [-0.57, 2.73] |
| Study 1b | Intercept | 9.88 | 1.46 | | 6.78 | <.001 | [6.98, 12.78] |
| | Total Knowledge | 1.59 | 0.72 | .29 | 2.23 | .029 | [0.17, 3.02] |
| | Task Skill | 0.79 | 0.49 | .21 | 1.63 | .107 | [-0.18, 1.76] |
| | Task Enjoyment | -0.20 | 0.48 | -.05 | -0.43 | .670 | [-1.15, 0.75] |
| | Task Familiarity | 0.33 | 0.44 | .08 | 0.75 | .459 | [-0.55, 1.20] |

***Total Knowledge Predicting Proofreading: Surface Errors***

**Table S12**

*Total Knowledge Predicting Surface-Level Proofreading Errors*

| | Predictors | *B* | *SE* | β | *t* | *p* | 95% CI |
|---|---|---|---|---|---|---|---|
| Full Sample | Intercept | 6.74 | 0.68 | | 9.94 | <.001 | [5.40, 8.07] |
| | Total Knowledge | 0.52 | 0.23 | .17 | 2.23 | .027 | [0.06, 0.98] |
| | Study | -0.06 | 0.30 | -.02 | -0.21 | .836 | [-0.65, 0.53] |
| | Task Skill | 0.68 | 0.33 | .19 | 2.07 | .040 | [0.03, 1.33] |
| | Task Enjoyment | 0.55 | 0.23 | .22 | 2.43 | .016 | [0.10, 1.01] |
| | Task Familiarity | -0.39 | 0.24 | -.13 | -1.64 | .104 | [-0.86, 0.08] |
| | Knowledge*Study | -0.43 | 0.23 | -.14 | -1.84 | .068 | [-0.88, 0.03] |
| Study 1a | Intercept | 6.40 | 0.88 | | 7.28 | <.001 | [4.65, 8.14] |
| | Total Knowledge | 0.96 | 0.33 | .29 | 2.87 | .005 | [0.30, 1.62] |
| | Task Skill | 0.24 | 0.42 | .07 | 0.58 | .564 | [-0.59, 1.07] |
| | Task Enjoyment | 0.75 | 0.30 | .31 | 2.52 | .014 | [0.16, 1.34] |
| | Task Familiarity | -0.55 | 0.32 | -.20 | -1.69 | .095 | [-1.19, 0.10] |
| Study 1b | Intercept | 7.37 | 1.05 | | 7.03 | <.001 | [5.29, 9.46] |
| | Total Knowledge | 0.09 | 0.32 | .03 | 0.28 | .781 | [-0.54, 0.72] |
| | Task Skill | 1.24 | 0.52 | .32 | 2.40 | .018 | [0.21, 2.26] |
| | Task Enjoyment | 0.31 | 0.35 | .12 | 0.88 | .383 | [-0.39, 1.01] |
| | Task Familiarity | -0.23 | 0.34 | -.08 | -0.68 | .497 | [-0.92, 0.45] |

***Total Knowledge Predicting Proofreading: Contextual Errors***

**Table S13**

*Total Knowledge Predicting Contextual-Level Proofreading Errors*

| Predictors | *B* | *SE* | β | *t* | *p* | 95% CI |
|---|---|---|---|---|---|---|
| Intercept | 2.96 | 0.56 | | 5.32 | <.001 | [1.86, 4.05] |
| Total Knowledge | 0.47 | 0.19 | .18 | 2.45 | .015 | [0.09, 0.84] |
| Study | -0.35 | 0.25 | -.10 | -1.42 | .158 | [-0.83, 0.14] |
| Task Skill | 0.42 | 0.27 | .14 | 1.57 | .119 | [-0.11, 0.95] |
| Task Enjoyment | 0.45 | 0.19 | .21 | 2.40 | .017 | [0.08, 0.82] |
| Task Familiarity | 0.10 | 0.19 | .04 | .52 | .604 | [-0.28, 0.49] |
| Knowledge*Study | -0.23 | 0.19 | -.09 | -1.20 | .232 | [-0.60, 0.15] |

**How do Eager and Vigilant Metamotivational Knowledge Relate to Performance?**

The main text presents the analyses for the relation between eager and vigilant knowledge and overall performance; brainstorming: number of ideas; brainstorming: originality; and proofreading: total errors. Here we present several additional analyses for full transparency.

***Eager and Vigilant Knowledge Predicting Overall Performance: Study Level Analyses***

As reported in the main text (see Table 6), we regressed participants' overall performance on study, task skill, task enjoyment, task familiarity, eager and vigilant knowledge, and the interactions between both types of knowledge and study. There was a marginal interaction between study and eager knowledge that paralleled the pattern found with total knowledge. Running the regression analysis separately for each study, eager knowledge emerged as a significant predictor of performance in Study 1a, but not Study 1b (see Table S14).

**Table S14**

*Regression Analyses Predicting Overall Task Performance From Eager and Vigilant Knowledge, Controlling for Study and Task Type, Skill, Enjoyment, and Familiarity*

| Predictors | | *b* | *SE* | β | *T* | *p* | 95% CI |
|---|---|---|---|---|---|---|---|
| Study 1a | Intercept | -0.24 | 0.18 | | -1.35 | .178 | [-0.59, 0.11] |
| | Eager Knowledge | 0.22 | 0.06 | .07 | 3.64 | <.001 | [0.10, 0.33] |
| | Vigilant Knowledge | 0.12 | 0.07 | .14 | 1.69 | .094 | [-0.04, 0.26] |
| | Task Type | 0.04 | 0.08 | .05 | 0.50 | .619 | [-0.17, 0.12] |
| | Task Skill | 0.10 | 0.06 | .15 | 1.70 | .092 | [-0.12, 0.20] |
| | Task Enjoyment | 0.05 | 0.07 | .07 | 0.76 | .451 | [-0.02, 0.22] |
| | Task Familiarity | 0.05 | 0.07 | .07 | 0.73 | .466 | [-0.08, 0.18] |
| | Eager*Task Type | 0.06 | 0.06 | .08 | 0.92 | .358 | [-0.06, 0.17] |
| | Vigilant*Task Type | 0.11 | 0.07 | .12 | 1.49 | .137 | [-0.04, 0.25] |
| Study 1b | Intercept | -0.25 | 0.19 | | -1.35 | .178 | [-0.62, 0.12] |
| | Eager Knowledge | 0.04 | 0.07 | .04 | .049 | .623 | [-0.10, 0.18] |
| | Vigilant Knowledge | 0.02 | 0.08 | .02 | 0.27 | .784 | [-0.13, 0.17] |
| | Task Type | 0.04 | 0.08 | .04 | 0.58 | .566 | [-0.11, 0.20] |
| | Task Skill | 0.17 | 0.09 | .18 | 1.30 | .060 | [-0.01, 0.35] |
| | Task Enjoyment | 0.08 | 0.06 | .12 | 1.29 | .198 | [-0.04, 0.20] |
| | Task Familiarity | 0.03 | 0.07 | .03 | 0.40 | .689 | [0.10, 0.16] |
| | Eager*Task Type | -0.001 | 0.07 | -.002 | -0.02 | .985 | [-0.14, 0.14] |
| | Vigilant*Task Type | 0.10 | 0.07 | .11 | 1.28 | .203 | [-0.05, 0.24] |

***Eager and Vigilant Knowledge Predicting Proofreading: Total Errors – Study Level Analysis***

As reported in the main text, we regressed participants' proofreading performance on study, task skill, task enjoyment, task familiarity, eager and vigilant knowledge, and the interactions between both types of knowledge and study. As indicated in Table 7 in the main text, there was a significant interaction between eager knowledge and study, indicating that the

effect of eager knowledge on proofreading performance was likely moderated by study. Conducting the regression analyses separately for each study, eager knowledge emerged as a significant predictor of proofreading performance in Study 1a ($b$ = 1.72, $p$ = .005), but not in Study 1b ($b$ = 0.05, $p$ = .927; see Table S15). Additionally, although there was no significant interaction between vigilant knowledge and study, we see the same pattern emerge such that vigilant knowledge emerged as a significant predictor in Study 1a ($b$ = 1.54, $p$ = .022), but not in Study 1b ($b$ = 0.63, $p$ = .242)

**Table S15**

*Eager and Vigilant Knowledge Predicting Total Proofreading Errors*

| | Predictors | *B* | *SE* | β | *t* | *p* | 95% CI |
|---|---|---|---|---|---|---|---|
| Study 1a | Intercept | 9.72 | 1.45 | | 6.71 | <.001 | [6.84, 12.61] |
| | Eager Knowledge | 1.72 | .60 | .35 | 2.88 | .005 | [0.53, 2.91] |
| | Vigilant Knowledge | 1.54 | .66 | .28 | 2.33 | .022 | [0.23, 2.85] |
| | Task Skill | .68 | .69 | .12 | .98 | .329 | [-0.70, 2.05] |
| | Task Enjoyment | 1.17 | .49 | .29 | 2.40 | .019 | [0.20, 2.14] |
| | Task Familiarity | -.36 | .53 | -.08 | -.67 | .504 | [-1.42, 0.70] |
| Study 1b | Intercept | 9.76 | 1.46 | | 6.682 | <.001 | [6.85, 12.70] |
| | Eager Knowledge | .05 | .52 | .010 | .092 | .927 | [-0.99, 1.08] |
| | Vigilant Knowledge | .63 | .53 | .128 | 1.178 | .242 | [-0.43, 1.68] |
| | Task Skill | 1.62 | .72 | .295 | 2.266 | .026 | [0.20, 3.05] |
| | Task Enjoyment | .80 | .49 | .210 | 1.643 | .104 | [-0.17, 1.77] |
| | Task Familiarity | -.26 | .48 | -.059 | -.543 | .589 | [-1.22, 0.70] |

***Eager and Vigilant Knowledge Predicting Proofreading: Surface Errors***

In this analysis, we examined the association of eager and vigilant knowledge separately with the detection of surface-level proofreading errors. Results revealed a significant main effect of vigilant knowledge on proofreading performance; there was no main effect of eager

knowledge. There was a significant interaction between study and eager knowledge that paralleled the pattern found with total knowledge (running the regression analysis separately for each study, eager knowledge emerged as a significant predictor of performance in Study 1a, but not Study 1b; see Table S16).

**Table S16**

*Eager and Vigilant Knowledge Predicting Surface-Level Proofreading Errors*

| | Predictors | *B* | *SE* | β | *t* | *p* | 95% CI |
|---|---|---|---|---|---|---|---|
| Full Sample | Intercept | 6.66 | 0.68 | | 9.81 | <.001 | [5.32, 8.00] |
| | Eager Knowledge | 0.36 | 0.26 | .12 | 1.38 | .169 | [-0.16, 0.88] |
| | Vigilant Knowledge | 0.68 | 0.28 | .21 | 2.45 | .015 | [0.13, 1.24] |
| | Study | -0.12 | 0.30 | -.03 | -0.39 | .700 | [-0.71, 0.48] |
| | Task Skill | 0.70 | 0.33 | .20 | 2.11 | .036 | [0.05, 1.34] |
| | Task Enjoyment | 0.56 | 0.23 | .22 | 2.46 | .015 | [0.11, 1.01] |
| | Task Familiarity | -0.42 | 0.24 | -.15 | -1.78 | .077 | [-0.89, 0.05] |
| | Eager*Study | -0.59 | 0.26 | -.19 | -2.26 | .025 | [-1.11, -0.08] |
| | Vigilant*Study | -0.25 | 0.28 | -.07 | -0.88 | .380 | [-0.80, 0.31] |
| Study 1a | Intercept | 6.38 | .89 | | 7.18 | <.001 | [4.61, 8.15] |
| | Eager Knowledge | .99 | .37 | .33 | 2.70 | .009 | [0.26, 1.72] |
| | Vigilant Knowledge | .91 | .41 | .28 | 2.25 | .027 | [0.11, 1.72] |
| | Task Skill | .23 | .42 | .07 | .54 | .590 | [-0.61, 1.07] |
| | Task Enjoyment | .75 | .30 | .31 | 2.51 | .014 | [0.15, 1.34] |
| | Task Familiarity | -.54 | .33 | -.20 | -1.66 | .100 | [-1.20, 0.11] |
| Study 1b | Intercept | 7.24 | 1.04 | | 6.95 | <.001 | [5.17, 9.32] |
| | Eager Knowledge | -.23 | .37 | -.07 | -.61 | .541 | [-0.96, 0.51] |
| | Vigilant Knowledge | .43 | .38 | .13 | 1.13 | .264 | [-0.33, 1.18] |
| | Task Skill | 1.27 | .51 | .33 | 2.49 | .015 | [0.25, 2.29] |
| | Task Enjoyment | .32 | .35 | .12 | .91 | .367 | [-0.38, 1.01] |
| | Task Familiarity | -.30 | .34 | -.10 | -.87 | .386 | [-0.98, 0.38] |

***Eager and Vigilant Knowledge Predicting Proofreading: Contextual Errors***

In this analysis, we examined the association of eager and vigilant knowledge separately with the detection of contextual-level proofreading errors. Results revealed a significant main effect of eager knowledge and a marginal main effect of vigilant knowledge on task performance. These results were consistent across Studies 1a and 1b (see Table S17).

**Table S17**

*Eager and Vigilant Knowledge Predicting Contextual-Level Proofreading Errors*

| Predictors | $B$ | $SE$ | β | $t$ | $p$ | $R^2$ |
|---|---|---|---|---|---|---|
| Intercept | 2.95 | 0.56 | | 5.25 | <.001 | [1.84, 4.06] |
| Eager Knowledge | 0.51 | 0.22 | .20 | 2.34 | .020 | [0.08, 0.94] |
| Vigilant Knowledge | 0.41 | 0.23 | .15 | 1.78 | .077 | [-0.05, 0.87] |
| Study | -0.33 | 0.25 | -.10 | -1.32 | .190 | [-0.82, 0.17] |
| Task Skill | 0.41 | 0.27 | .14 | 1.51 | .134 | [-0.13, 0.95] |
| Task Enjoyment | 0.45 | 0.19 | .21 | 2.39 | .018 | [0.08, 0.82] |
| Task Familiarity | 0.11 | 0.12 | .05 | 0.55 | .583 | [-0.28, 0.50] |
| Eager*Study | -0.23 | 0.22 | -.09 | -1.06 | .292 | [-0.66, 0.20] |
| Vigilant*Study | -0.22 | 0.23 | -.08 | -0.94 | .349 | [-0.68, 0.24] |

## Study 2 Supplemental Materials

### Regulatory Focus Knowledge Assessment

Eager Task Descriptions:

1. Your goal is to be as creative as possible by seizing opportunities to take the ordinary and innovate.
2. Your goal is to imagine a future no one has seen before by seeing possibilities and occasions for advancement.
3. Imagine that you work for an advertising firm and are responsible for pitching a new ad campaign to a major client. The client wants the campaign to be witty, innovative, and completely novel—unlike anything that's been seen on television before.

Vigilant Task Descriptions:

1. Your goal is to be as accurate as possible by making sure to avoid lurking errors and pitfalls.
2. Your goal is to be precise and make sure that you don't make a wrong turn in figuring out the right next step.
3. Imagine you are working in a plant that deals with volatile and potentially dangerous materials. Your job is to figure out the best system for managing workers who inspect products to ensure that they adhere to all safety standards.

Promotion Recall Activities:

1. Please write about a time in the past when you felt you made progress toward being successful in life.
2. Please write about a time in the past when compared to most people you were able to get what you wanted out of life.
3. Please write about a time in the past when trying to achieve something important to you, you performed as well as you ideally would have liked to.
4. Please write about your hopes and aspirations as a child. What accomplishments did you ideally want to meet when you were a child?

Prevention Recall Activities:

1. Please write about a time in the past when being careful enough avoided getting you into trouble.
2. Please write about a time in the past when you stopped yourself from acting in a way that your parents would have considered objectionable.
3. Please write about a time in the past when you were careful not to get on your parents' nerves.
4. Please write about your duties and obligations as a child. What responsibilities did you think you ought to meet when you were a child?

**Additional Measures**

Studies 2a and 2b were larger online studies that contained additional materials beyond the primary construct of interest reported in the main text. Study 2a and 2b also administered a knowledge assessment that measured the extent to which people recognize the benefits of high-level vs. low-level construal for tasks that benefit from these motivational states. In addition, Study 2a also presented participants with a knowledge assessment that measured whether people appreciate the motivational benefits of positive and negative feedback in goal pursuit. Given that these measures are not the primary focus of the current paper, we do not report the results for these measures.

## Study 2 Supplemental Analyses

### Using a Subset of the Exclusion Criteria from the Main Text

The main text excluded across all analyses: (1) participants who reported that they were not paying attention during the study (i.e., reported being "very" or "extremely" distracted or taking the study "not at all" or "a little" seriously; *n* = 122), (2) those who reported they were not fluent in English (*n* = 84), (3) those who did not consent to share their grades with us (*n* = 32), (4) those whose academic records we could not retrieve (*n* = 5), (5) those who did not report a high school GPA (*n* = 38), and (6) those with an unusual degree of missing responses (> 50%) in the knowledge assessment (*n* = 2). This resulted in a final *N* = 368, or 62% of the original sample of *N* = 592. Given this unexpectedly low retention of the original sample, we conduct and report analyses on the following pages that use only a subset of the above exclusion criteria. We exclude: (1) those who did not consent to share their grades with us (*n* = 32), (2) those whose academic records we could not retrieve (*n* = 5), (3) those who did not report a high school GPA (*n* = 38), and (4) those with more than one missing response in the knowledge assessment (*n* = 4). As a result, the analyses reported below have a final *N* = 520 (88% of the original sample). As noted in the main text, the pattern of results reported here are generally consistent with the results in the paper and are stronger, perhaps due to the increase in statistical power.

### Metamotivational Knowledge of Regulatory Focus

To examine whether participants understand how to create regulatory focus task-motivation fit, we submitted their usefulness ratings to a 2 (task: eager vs. vigilant) x 2 (recall activity: promotion vs. prevention) repeated measures ANOVA. Results revealed a main effect of task such that participants provided higher usefulness ratings for vigilant tasks (*M* = 4.57, *SD* = .72) than for eager tasks (*M* = 4.30, *SD* = .78), $F(1, 519) = 140.37$, $p < .001$, $\eta_p^2 = .21$. There

was also a main effect of recall activity such that participants reported higher usefulness ratings for promotion recall activities (*M* = 4.61, *SD* = .71) than for prevention recall activities (*M* = 4.26, *SD* = .81), *F*(1, 519) = 186.04, *p* < .001, $\eta_p^2$ = .26. Importantly, as expected, results also revealed a significant task x recall activity interaction, *F*(1, 519) = 462.85, *p* < .001, $\eta_p^2$ = .47.

To decompose this interaction, we first conducted simple slopes as a function of recall activity. Participants rated that the promotion recall activities would be more useful for eager tasks (*M* = 4.84, *SD* = .74) vs. vigilant tasks (*M* = 4.38, *SD* = .83), *t*(519) = 15.58, *p* < .001, *d* = .69. Participants also rated that prevention recall activities would be more useful for vigilant tasks (*M* = 4.76, *SD* = .80) vs. eager tasks (*M* = 3.75, *SD* = 1.15), *t*(519) = 20.09, *p* < .001, *d* = .91. Next, we conducted simple slopes as a function of task. As expected, within eager tasks, participants rated that promotion recall activities would be more useful than prevention recall activities, *t*(519) = 21.67, *p* < .001, *d* = .99. By contrast, within vigilant tasks, participants rated that prevention recall activities would be more useful than promotion recall activities, *t*(519) = 11.11, *p* < .001, *d* = .49. In sum, these findings suggest that students recognize regulatory focus task-motivation fit.

***Predicting grades in PSYCH 1100 from knowledge of how to create task-motivation fit***

Next, we conducted a regression analysis to examine whether students' knowledge of how to create regulatory focus task-motivation fit predicts their final grade in PSYCH 1100, above and beyond other variables that traditionally predict grades and other covariates (see Table S18 for zero-order correlations). To prepare the data, we converted letter grades (*M* = 3.29, *SD* = .85) to a 4-point scale (A = 4.0, A- = 3.7, B+ = 3.4, etc.). We also created an overall metamotivational knowledge index (*M* = 1.46, *SD* = 1.55): ([usefulness of promotion recall activities for eager tasks – usefulness of prevention recall activities for eager tasks] + [usefulness

of prevention recall activities for vigilant tasks – usefulness of promotion recall activities for vigilant tasks]). Continuous predictors were standardized in all regression analyses to allow for meaningful interpretations in the predicted change in grades as a function of different variables.

**Table S18**

*Zero-Order Correlations for Study 2 (Subset of the Exclusion Criteria from the Main Text)*

| | 1 | 2 | 3 | 4 | 5 | 6 | 7 | 8 | 9 |
|---|---|---|---|---|---|---|---|---|---|
| 1. Final Grade in PSYCH 1100 | - | | | | | | | | |
| 2. Total Knowledge | .32*** | - | | | | | | | |
| 3. Eager Knowledge | .29*** | .88*** | - | | | | | | |
| 4. Vigilant Knowledge | .22*** | .71*** | .28*** | - | | | | | |
| 5. High School GPA | .29*** | .14** | .14** | .08 | - | | | | |
| 6. Academic Motivation | .21** | .08 | .10* | .03 | .05* | - | | | |
| 7. Academic Self-Concept | .38*** | .14* | .10* | .14* | .17*** | .15** | - | | |
| 8. Gender (higher = female) | .14* | .05 | .11* | -.04 | .12** | .21** | -.01 | - | |
| 9. Age | -.23*** | -.002 | .03 | -.04 | -.29*** | -.18** | -.07 | -.22*** | - |
| 10. Major (higher = psych major) | .004 | -.06 | -.05 | -.05 | -.06 | .13** | .01 | .10* | -.07 |

*Note:* *$p < .05$, **$p < .01$, ***$p < .001$.

We regressed students' grades on total knowledge, high school GPA, academic motivation, academic self-concept, gender (-0.5 = male, 0.5 = female or unidentified), age, major (-0.5 = other major, 0.5 = psychology major), study (-0.5 = Study 2a, 0.5 = Study 2b), and total knowledge x study. This model was significant, $F(9, 516) = 25.31$, $p < .001$ (see Table S19). High school GPA, $b = .13$, $SE = .03$, $t(516) = 3.86$, $p < .001$, academic self-concept, $b = .27$, $SE = .03$, $t(516) = 8.11$, $p < .001$, and age, $b = -.12$, $SE = .03$, $t(516) = 3.52$, $p < .001$, significantly predicted grades. As expected, students' knowledge of how to create task-motivation fit significantly predicted their final grades in PSYCH 1100, $b = .21$, $SE = .03$, $t(516) = 6.55$, $p < .001$. Study did not significantly moderate this effect, $p = .28$. Different from the main text, academic motivation significantly predicted grades, $b = .07$, $SE = .03$, $t(516) = 2.10$, $p = .037$, and gender marginally predicted grades, $b = .12$, $SE = .07$, $t(516) = 1.81$, $p = .071$. All other predictors were non-significant, *p*'s > .61.

**Table S19**

*Regression Analysis Predicting Final Grades – Total Knowledge (Study 2)*

| Predictors | *b* | *SE* | β | *t* | *p* | 95% CI |
|---|---|---|---|---|---|---|
| Intercept | 3.29 | 0.05 | | 66.64 | < .001 | [3.20, 3.39] |
| Total Knowledge | 0.21 | 0.03 | .24 | 6.55 | < .001 | [.15, .28] |
| High School GPA | 0.13 | 0.03 | .15 | 3.86 | < .001 | [.06, .20] |
| Academic Motivation | 0.07 | 0.03 | .08 | 2.10 | .037 | [.004, .14] |
| Academic Self-Concept | 0.27 | 0.03 | .31 | 8.11 | < .001 | [.20, .33] |
| Gender | 0.12 | 0.07 | .07 | 1.81 | .071 | [-.01, .25] |
| Age | -0.12 | 0.03 | -.14 | -3.52 | < .001 | [-.19, -.05] |
| Major | -0.01 | 0.10 | -.01 | -0.14 | .889 | [-.21, .18] |
| Study | 0.03 | 0.06 | -.02 | 0.52 | .607 | [-.09, .16] |
| Total Knowledge * Study | -0.07 | 0.06 | -.04 | -1.09 | .278 | [-.19, .06] |

Next, we examined what kind of knowledge predicts grades. We regressed students' grades on eager knowledge, vigilant knowledge, high school GPA, academic motivation, academic self-concept, gender (-0.5 = male, 0.5 = female or unidentified), age, major (-0.5 = other major, 0.5 = psychology major), study (-0.5 = Study 2a, 0.5 = Study 2b), eager knowledge x study, and vigilant knowledge x study. This model was significant, $F(11, 516) = 20.67$, $p < .001$ (see Table S20). Results revealed that eager knowledge significantly predicted grades, $b = .17$, $SE = .03$, $t(516) = 4.97$, $p < .001$. Additionally, vigilant knowledge significantly predicted grades, $b = .09$, $SE = .03$, $t(516) = 2.63$, $p = .009$. Thus, students' knowledge of how to create task-motivation fit—both eager and vigilant knowledge—predicts their final grades in PSYCH 1100.

**Table S20**

*Regression Analysis Predicting Final Grades – Eager and Vigilant Knowledge (Study 2)*

| Predictors | *b* | *SE* | β | *t* | *p* | 95% CI |
|---|---|---|---|---|---|---|
| Intercept | 3.29 | 0.05 | | 66.34 | < .001 | [3.20, 3.39] |
| Eager Knowledge | 0.17 | 0.03 | .20 | 4.97 | < .001 | [.10, .24] |
| Vigilant Knowledge | 0.09 | 0.03 | .10 | 2.63 | .009 | [.02, .15] |
| High School GPA | 0.13 | 0.03 | .15 | 3.82 | < .001 | [.06, .20] |
| Academic Motivation | 0.07 | 0.03 | .08 | 2.07 | .039 | [.004, .14] |
| Academic Self-Concept | 0.27 | 0.03 | .31 | 8.12 | < .001 | [.20, .33] |
| Gender | 0.12 | 0.07 | .07 | 1.73 | .085 | [-.02, .25] |
| Age | -0.12 | 0.03 | -.14 | -3.54 | < .001 | [-.19, -.05] |
| Major | -0.01 | 0.10 | -.01 | -0.14 | .887 | [-.21, .18] |
| Study | 0.03 | 0.06 | .02 | 0.54 | .590 | [-.09, .16] |
| Eager Knowledge * Study | -0.05 | 0.07 | -.03 | -0.76 | .488 | [-.18, .08] |
| Vigilant Knowledge * Study | -0.03 | 0.07 | -.02 | -0.49 | .622 | [-.16, .10] |

**Including Participants with Unusual Degrees of Missing Responses (> 50%)**

As previously mentioned, the main text excluded across all analyses: (1) participants who reported that they were not paying attention during the study (i.e., reported being "very" or "extremely" distracted or taking the study "not at all" or "a little" seriously; *n* = 122), (2) those who reported they were not fluent in English (*n* = 84), (3) those who did not consent to share their grades with us (*n* = 32), (4) those whose academic records we could not retrieve (*n* = 5), (5) those who did not report a high school GPA (*n* = 38), and (6) those with an unusual degree of missing responses (> 50%) in the knowledge assessment (*n* = 2). Given that exclusion criterion #6 was not an *a priori* decision, we report analyses that only use exclusion criteria 1 – 5 for transparency. These analyses include one additional participant, for a sample of *N* = 369 (62% of the original sample).

**Metamotivational Knowledge of Regulatory Focus**

To examine whether participants understand how to create task-motivation fit, we submitted their usefulness ratings to a 2 (task: eager vs. vigilant) x 2 (recall activity: promotion vs. prevention) repeated measures ANOVA. Results revealed a main effect of task such that participants provided higher usefulness ratings for vigilant tasks (*M* = 4.56, *SD* = .67) than for eager tasks (*M* = 4.19, *SD* = .74), $F(1, 368) = 166.14$, $p < .001$, $\eta_p^2 = .31$. There was also a main effect of recall activity such that participants reported higher usefulness ratings for promotion recall activities (*M* = 4.59, *SD* = .68) than for prevention recall activities (*M* = 4.16, *SD* = .75), $F(1, 368) = 187.43$, $p < .001$, $\eta_p^2 = .34$. Importantly, as expected, results also revealed a significant task x recall activity interaction, $F(1, 368) = 494.96$, $p < .001$, $\eta_p^2 = .57$.

To decompose this interaction, we first tested simple effects as a function of recall activity. As expected, participants rated that the promotion recall activities would be more useful

for eager tasks (*M* = 4.86, *SD* = .72) vs. vigilant tasks (*M* = 4.32, *SD* = .80), *t*(368) = 15.58, *p* < .001, *d* = .81. Participants also rated that prevention recall activities would be more useful for vigilant tasks (*M* = 4.79, *SD* = .77) vs. eager tasks (*M* = 3.53, *SD* = 1.10), *t*(368) = 20.91, *p* < .001, *d* = 1.11. Next, we conducted simple slopes as a function of task. As expected, within eager tasks, participants rated that promotion recall activities would be more useful than prevention recall activities, *t*(368) = 22.69, *p* < .001, *d* = 1.22. By contrast, within vigilant tasks, participants rated that prevention recall activities would be more useful than promotion recall activities, *t*(368) = 11.10, *p* < .001, *d* = .57. In sum, students recognize how to create regulatory focus task-motivation fit.

***Predicting grades in PSYCH 1100 from knowledge of how to create task-motivation fit***

Next, we conducted a regression analysis to examine whether students' knowledge of how to create regulatory focus task-motivation fit predicts their final grade in PSYCH 1100, above and beyond other variables that traditionally predict grades and other covariates (see Table S21 for zero-order correlations). To prepare the data, we converted letter grades (*M* = 3.40, *SD* = .77) to a 4-point scale (A = 4.0, A- = 3.7, B+ = 3.4, etc.). We also created an overall metamotivational knowledge index (*M* = 1.81, *SD* = 1.56) as in Study 1 ([usefulness of promotion recall activities for eager tasks – usefulness of prevention recall activities for eager tasks] + [usefulness of prevention recall activities for vigilant tasks – usefulness of promotion recall activities for vigilant tasks]). Continuous predictors were standardized in all regression analyses to allow for meaningful interpretations in the predicted change in grades as a function of different variables.

**Table S21**

*Zero-Order Correlations for Study 2 (Participants with Unusual Degrees of Missing Responses)*

| | 1 | 2 | 3 | 4 | 5 | 6 | 7 | 8 | 9 |
|---|---|---|---|---|---|---|---|---|---|
| 1. Final Grade in PSYCH 1100 | - | | | | | | | | |
| 2. Total Knowledge | .31*** | - | | | | | | | |
| 3. Eager Knowledge | .28*** | .86*** | - | | | | | | |
| 4. Vigilant Knowledge | .19*** | .72*** | .26*** | - | | | | | |
| 5. High School GPA | .33*** | .16** | .16** | .08 | - | | | | |
| 6. Academic Motivation | .15** | .02 | .03 | -.01 | .11* | - | | | |
| 7. Academic Self-Concept | .41*** | .13* | .09 | .12* | .19*** | .17** | - | | |
| 8. Gender (higher = female) | .13* | -.01 | .05 | -.09 | .16** | .17** | -.004 | - | |
| 9. Age | -.23*** | .01 | .02 | -.01 | -.35*** | -.16** | -.07 | -.21*** | - |
| 10. Major (higher = psych major) | -.004 | -.04 | -.02 | -.05 | .01 | .15** | -.04 | .10* | -.04 |

*Note:* *$p$ < .05, **$p$ < .01, ***$p$ < .001.

We regressed students' grades on total knowledge, high school GPA, academic motivation, academic self-concept, gender (-0.5 = male, 0.5 = female or unidentified), age, major (-0.5 = other major, 0.5 = psychology major), study (-0.5 = Study 2a, 0.5 = Study 2b), and total knowledge x study. This model was significant, $F(9, 359) = 17.68$, $p < .001$ (see Table S22). High school GPA, $b = .13$, $SE = .04$, $t(359) = 3.57$, $p < .001$, academic self-concept, $b = .25$, $SE = .04$, $t(359) = 7.13$, $p < .001$, and age, $b = -.09$, $SE = .04$, $t(359) = 2.55$, $p = .011$, significantly predicted grades. As expected, students' knowledge of how to create task-motivation fit significantly predicted their final grades in PSYCH 1100, $b = .18$, $SE = .04$, $t(359) = 5.29$, $p < .001$. Study did not significantly moderate this effect, $p = .57$. All other predictors were non-significant, $p$'s > .13.

**Table S22**

*Regression Analysis Predicting Final Grades – Total Knowledge (Study 2)*

| Predictors | *b* | *SE* | β | *t* | *p* | 95% CI |
|---|---|---|---|---|---|---|
| Intercept | 3.40 | 0.06 | | 62.12 | < .001 | [3.29, 3.50] |
| Total Knowledge | 0.18 | 0.04 | .24 | 5.29 | < .001 | [.12, .25] |
| High School GPA | 0.13 | 0.04 | .17 | 3.57 | < .001 | [.06, .21] |
| Academic Motivation | 0.03 | 0.04 | .04 | 0.90 | .367 | [-.04, .10] |
| Academic Self-Concept | 0.25 | 0.04 | .33 | 7.13 | < .001 | [.18, .32] |
| Gender | 0.11 | 0.07 | .07 | 1.52 | .130 | [-.03, .25] |
| Age | -0.09 | 0.04 | -.12 | -2.55 | .011 | [-.17, -.02] |
| Major | -0.004 | 0.11 | -.002 | -0.04 | .968 | [-.22, .21] |
| Study | -0.02 | 0.07 | -.01 | -0.33 | .745 | [-.16, .11] |
| Total Knowledge * Study | -0.04 | 0.07 | -.03 | -0.57 | .569 | [-.17, .10] |

Next, we examined what kind of knowledge predicts grades. To do so, we separated the total knowledge index into two indices: eager knowledge (usefulness of promotion recall activities for eager tasks – usefulness of prevention recall activities for eager tasks) and vigilant knowledge (usefulness of prevention recall activities for vigilant tasks – usefulness of promotion recall activities for vigilant tasks). We regressed students' grades on eager knowledge, vigilant knowledge, high school GPA, academic motivation, academic self-concept, gender (-0.5 = male, 0.5 = female or unidentified), age, major (-0.5 = other major, 0.5 = psychology major), study (-0.5 = Study 2a, 0.5 = Study 2b), eager knowledge x study, and vigilant knowledge x study. This model was significant, $F(11, 357) = 14.48$, $p < .001$ (see Table S23). Results revealed that eager knowledge significantly predicted grades, $b = .15$, $SE = .04$, $t(357) = 4.29$, $p < .001$. Additionally, vigilant knowledge marginally predicted grades, $b = .07$, $SE = .04$, $t(357) = 1.96$, $p = .051$. Thus, students' knowledge of how to create task-motivation fit—specifically both eager and vigilant knowledge—predicts their final grades in PSYCH 1100.

**Table S23**

*Regression Analysis Predicting Final Grades – Eager and Vigilant Knowledge (Study 2)*

| Predictors | *b* | *SE* | β | *t* | *p* | 95% CI |
|---|---|---|---|---|---|---|
| Intercept | 3.40 | 0.06 | | 61.84 | < .001 | [3.29, 3.50] |
| Eager Knowledge | 0.15 | 0.04 | .20 | 4.29 | < .001 | [.08, .23] |
| Vigilant Knowledge | 0.07 | 0.04 | .09 | 1.96 | .051 | [-.0002, .14] |
| High School GPA | 0.13 | 0.04 | .17 | 3.52 | < .001 | [.06, .21] |
| Academic Motivation | 0.03 | 0.04 | .04 | 0.88 | .378 | [-.04, .10] |
| Academic Self-Concept | 0.25 | 0.04 | .33 | 7.15 | < .001 | [.18, .32] |
| Gender | 0.10 | 0.07 | .07 | 1.40 | .162 | [-.04, .24] |
| Age | -0.10 | 0.04 | -.13 | -2.58 | .010 | [-.17, -.02] |
| Major | -0.01 | 0.11 | -.002 | -0.05 | .962 | [-.22, .21] |
| Study | -0.02 | 0.07 | -.01 | -0.29 | .772 | [-.16, .12] |
| Eager Knowledge * Study | -0.03 | 0.07 | -.02 | -0.37 | .715 | [-.17, .11] |
| Vigilant Knowledge * Study | -0.02 | 0.07 | -.01 | -0.29 | .769 | [-.16, .12] |

**Additional Analyses Including Extent to Which Participants Took Study Seriously**

We conducted additional analyses that included the item in which participants indicated the extent to which they took the study seriously, which could be considered a proxy for conscientiousness. Below, we report analyses with the sample reported in the main text (*N* = 368). Additionally, given that the exclusion criteria to derive the final *N* in the main text involved the item regarding how seriously participants took the study, we also reported analyses with the larger sample that did not use this item as a means for exclusion (specific exclusion criteria reported on page 37 of the SOM; *N* = 520). We found that the "serious" item was positively correlated with metamotivational knowledge and course grades (see Tables S24 & S27). However, even when it was included as a covariate in our models, the relation between metamotivational knowledge and course grades remained significant (see Tables S25-S26, S28-S29).

***Sample Reported in Main Text (N = 368)***

**Table S24**

*Zero-Order Correlations for "Serious" Item (N = 368)*

| | Serious Item |
|---|---|
| Final Grade in PSYCH 1100 | .17** |
| Total Knowledge | .29*** |
| Eager Knowledge | .31*** |
| Vigilant Knowledge | .14** |
| Academic Self-Concept | .19*** |
| Academic Motivation | .15** |

*Note:* *$p < .05$, **$p < .01$, ***$p < .001$

**Table S25**

*Regression Analysis Predicting Final Grades – Total Knowledge and Serious Item (N = 368)*

| Predictors | *b* | *SE* | β | *t* | *p* | 95% CI |
|---|---|---|---|---|---|---|
| Intercept | 3.40 | 0.06 | | 62.00 | <0.001 | [3.29, 3.50] |
| Serious Item | 0.02 | 0.04 | .02 | 0.41 | 0.680 | [-0.06, 0.09] |
| Total Knowledge | 0.18 | 0.04 | .23 | 4.97 | <0.001 | [.12, .25] |
| High School GPA | 0.13 | 0.04 | .17 | 3.51 | 0.000 | [.06, .21] |
| Academic Motivation | 0.03 | 0.04 | .04 | 0.81 | 0.418 | [-.04, .10] |
| Academic Self-Concept | 0.25 | 0.04 | .32 | 7.02 | 0.000 | [.18, .32] |
| Gender | 0.10 | 0.07 | .07 | 1.46 | 0.145 | [-.04, .24] |
| Age | -0.10 | 0.04 | -.13 | -2.58 | 0.010 | [-.17, -.02] |
| Major | -0.002 | 0.11 | -.001 | -0.02 | 0.983 | [-.22, .21] |
| Study | -0.02 | 0.07 | -.01 | -0.28 | 0.777 | [-.16, .12] |
| Total Knowledge * Study | -0.04 | 0.07 | -.03 | -0.57 | 0.571 | [-.17, .10] |

**Table S26**

*Regression Analysis Predicting Final Grades – Eager and Vigilant Knowledge and Serious Item (N = 368)*

| Predictors | *b* | *SE* | β | *t* | *p* | 95% CI |
|---|---|---|---|---|---|---|
| Intercept | 3.40 | 0.06 | | 61.72 | <0.001 | [3.29, 3.50] |
| Serious Item | 0.01 | 0.04 | .02 | 0.33 | 0.743 | [-0.06, 0.09] |
| Eager Knowledge | 0.15 | 0.04 | .20 | 4.06 | <0.001 | [.08, .23] |
| Vigilant Knowledge | 0.07 | 0.04 | .09 | 1.94 | 0.053 | [-.001, .14] |
| High School GPA | 0.13 | 0.04 | .17 | 3.46 | 0.001 | [.06, .21] |
| Academic Motivation | 0.03 | 0.04 | .04 | 0.81 | 0.419 | [-.04, .10] |
| Academic Self-Concept | 0.25 | 0.04 | .33 | 7.05 | <0.001 | [.18, .32] |
| Gender | 0.10 | 0.07 | .06 | 1.35 | 0.177 | [-.04, .24] |
| Age | -0.10 | 0.04 | -.13 | -2.60 | 0.010 | [-.17, -.02] |
| Major | -0.003 | 0.11 | .001 | -0.03 | 0.976 | [-.22, .21] |
| Study | -0.02 | 0.07 | -.01 | -0.25 | 0.801 | [-.15, .12] |
| Eager Knowledge * Study | -0.03 | 0.07 | -.02 | -0.36 | 0.716 | [-.17, .12] |
| Vigilant Knowledge * Study | -0.02 | 0.07 | -.01 | -0.31 | 0.760 | [-.16, .12] |

***Sample Reported in the SOM (N = 520)***

**Table S27**

*Zero-Order Correlations for "Serious" Item (N = 520)*

| | Serious Item |
|---|---|
| Final Grade in PSYCH 1100 | .07 |
| Total Knowledge | .37*** |
| Eager Knowledge | .36*** |
| Vigilant Knowledge | .21*** |
| Academic Self-Concept | .15*** |
| Academic Motivation | .20*** |

*Note:* **p* < .05, ***p* < .01, ****p* < . 001

**Table S28**

*Regression Analysis Predicting Final Grades – Total Knowledge and Serious Item (N = 520)*

| Predictors | *b* | *SE* | β | *t* | *p* | 95% CI |
|---|---|---|---|---|---|---|
| Intercept | 3.28 | 0.05 | | 66.70 | <0.001 | [3.18, 3.38] |
| Serious Item | -0.10 | 0.04 | -.11 | -2.77 | <0.001 | [-0.17, -0.03] |
| Total Knowledge | 0.24 | 0.03 | .29 | 7.14 | <0.001 | [.18, .31] |
| High School GPA | 0.13 | 0.03 | .16 | 3.94 | <0.001 | [.07, .20] |
| Academic Motivation | 0.09 | 0.03 | .10 | 2.55 | 0.011 | [.02, .15] |
| Academic Self-Concept | 0.27 | 0.03 | .32 | 8.34 | <0.001 | [.21, .34] |
| Gender | 0.13 | 0.07 | .08 | 1.99 | 0.047 | [.002, .26] |
| Age | -0.14 | 0.04 | -.13 | -3.25 | 0.001 | [-.22, -.06] |
| Major | -0.02 | 0.10 | -.01 | -0.22 | 0.828 | [-.22, .17] |
| Study | 0.03 | 0.06 | .02 | 0.42 | 0.675 | [-.10, .15] |
| Total Knowledge * Study | -0.08 | 0.06 | -.05 | -1.26 | 0.209 | [-.20, .04] |

**Table S29**

*Regression Analysis Predicting Final Grades – Eager and Vigilant Knowledge and Serious Item (N = 520)*

| Predictors | *b* | *SE* | β | *t* | *p* | 95% CI |
|---|---|---|---|---|---|---|
| Intercept | 3.28 | 0.05 | | 66.43 | <0.001 | [3.18, 3.38] |
| Serious Item | -0.10 | 0.04 | -.11 | -2.81 | 0.005 | [-0.17, -0.03] |
| Eager Knowledge | 0.20 | 0.03 | .23 | 5.61 | <0.001 | [0.13, 0.23] |
| Vigilant Knowledge | 0.10 | 0.03 | .12 | 2.99 | 0.003 | [0.03, .16] |
| High School GPA | 0.13 | 0.03 | .15 | 3.89 | <0.001 | [0.07, .20] |
| Academic Motivation | 0.09 | 0.03 | .10 | 2.53 | 0.012 | [0.02, 0.15] |
| Academic Self-Concept | 0.27 | 0.03 | .32 | 8.35 | <0.001 | [0.21, 0.34] |
| Gender | 0.13 | 0.07 | .07 | 1.89 | 0.059 | [-0.01, 0.26] |
| Age | -0.14 | 0.04 | -.13 | -3.29 | 0.001 | [-0.22, -0.06] |
| Major | -0.02 | 0.10 | -.01 | -0.22 | 0.823 | [-0.22, 0.17] |
| Study | 0.03 | 0.06 | .02 | 0.45 | 0.654 | [-0.10, 0.15] |
| Eager Knowledge * Study | -0.06 | 0.07 | -.04 | -0.91 | 0.366 | [-0.19, 0.07] |
| Vigilant Knowledge * Study | -0.04 | 0.07 | -.02 | -0.54 | 0.586 | [-0.16, 0.09] |

**Pilot Study Materials (Follow up for Study 2 Presented in Study 2 Introduction)**

In this survey, we will ask you about your experience in PSYCH 1100. Please take a moment to think about your PSYCH 1100 course. Think about the exams, assignments, lectures, research participation, etc. We are interested in how students succeed in PSYCH 1100. Specifically, we are interested in what strategies you think are useful for doing well in PSYCH 1100. On the next page, we will describe some of these strategies.

*Strategies (counterbalanced)*
Strategy A: Think about your hopes and aspirations, focus on gaining points, think about achieving a good grade.
Strategy B: Think about your duties and obligations, focus on not losing points, think about avoiding a bad grade.
Strategy C: Take a step back and focus on the big picture, zoom out to have a broad perspective, see the forest instead of the trees.
Strategy D: Get in the zone and focus on the details, zoom in to have a narrow perspective, see the trees instead of the forest.

*The following questions used these scale anchors: (extremely unhelpful – extremely helpful)*
To what extent is Strategy A helpful for doing well in PSYCH 1100?
To what extent is Strategy B helpful for doing well in PSYCH 1100?
To what extent is Strategy C helpful for doing well in PSYCH 1100?
To what extent is Strategy D helpful for doing well in PSYCH 1100?

Now, please consider all the parts of PSYCH 1100 that count for a grade.

- Exams (Exam I, Exam II, Final Exam)
- Section Points
- REP (Research Experience Program) - participation or paper
- Reflection Paper

For your section of PSYCH 1100, what assignments or activities count toward your **Section Points?** (for example, your section might require you to do in-class activities or writing assignments)
[open-ended text box]

*The following questions used these scale anchors: (extremely unhelpful – extremely helpful)*
To what extent is Strategy A helpful for doing well **on your exams in PSYCH 1100?**
To what extent is Strategy A helpful for doing well **on requirements for Section Points in PSYCH 1100?**
To what extent is Strategy A helpful for doing well **on requirements for REP in PSYCH 1100?**
To what extent is Strategy A helpful for doing well **on the Reflection Paper in PSYCH 1100?**

To what extent is Strategy B helpful for doing well **on your exams in PSYCH 1100?**
To what extent is Strategy B helpful for doing well **on requirements for Section Points in PSYCH 1100?**
To what extent is Strategy B helpful for doing well **on requirements for REP in PSYCH 1100?**

To what extent is Strategy B helpful for doing well **on the Reflection Paper in PSYCH 1100?**

To what extent is Strategy C helpful for doing well **on your exams in PSYCH 1100?**
To what extent is Strategy C helpful for doing well **on requirements for Section Points in PSYCH 1100?**
To what extent is Strategy C helpful for doing well **on requirements for REP in PSYCH 1100?**
To what extent is Strategy C helpful for doing well **on the Reflection Paper in PSYCH 1100?**

To what extent is Strategy D helpful for doing well **on your exams in PSYCH 1100?**
To what extent is Strategy D helpful for doing well **on requirements for Section Points in PSYCH 1100?**
To what extent is Strategy D helpful for doing well **on requirements for REP in PSYCH 1100?**
To what extent is Strategy D helpful for doing well **on the Reflection Paper in PSYCH 1100?**

## Pilot Study Results

In the main text, the means and statistics reported refer to the weighted ratings (usefulness ratings multiplied by percentage of course grade). Results using the unweighted, overall course ratings also suggest that participants viewed PSYCH 1100 as a course that benefitted more from engaging with a promotion focus ($M$ = 5.63, $SD$ = 1.44) than a prevention focus ($M$ = 5.11, $SD$ = 1.44), $t$(108) = 3.05, $p$ = .003. Both responses were once again significantly above the midpoint, *promotion:* $t$(108) = 14.50, $p$ < .001; *prevention:* $t$(108) = 8.04, $p$ <. 001, suggesting that students perceived both promotion and prevention motivation as beneficial for overall course performance.

Examining each course component individually, a promotion focus was seen as more helpful than a prevention focus for exams, $t$(108) = 3.67, $p$ < .001, and the reflection paper, $t$(108) = 2.62, $p$ = .010; there was no significant difference for section points, $t$(108) = .325, $p$ = .746, or research participation, $t$(108) = .174, $p$ = .862 (see Table S30).

**Table S30**

*Means and Standard Deviations for Ratings of Promotion and Prevention Focus for Each Course Component*

| | *M* (*SD*) | |
|---|---|---|
| | Promotion Focus | Prevention Focus |
| Exams (70.42%) | 5.79 (1.19) | 5.17 (1.47) |
| Section Points (18.31%) | 5.33 (1.23) | 5.28 (1.28) |
| Research Participation (REP; 9.86%) | 5.24 (1.28) | 5.21 (1.43) |
| Reflection Paper (1.41%) | 5.50 (1.19) | 5.09 (1.46) |

# Meta-Analysis

We examined the relationship between metamotivational knowledge and performance in four studies. Although most of the results were in the predicted direction and significant, there were instances (e.g., Study 1b) in which the results were non-significant. Thus, to determine the robustness and better understand the strength of the relationship between metamotivational knowledge and performance, we meta-analyzed the four samples included in the main text (Studies 1a and 1b, Studies 2a and 2b).

## Method

We employed a bare-bones meta-analysis as outlined by Schmidt and Hunter (2015). Specifically, we meta-analyzed the raw correlations between total knowledge, eager knowledge, vigilant knowledge and performance using the *psychmeta* package in R (Dahlke & Wiernik, 2019), which conducts bare-bones meta-analysis to compute a mean sample-weighted correlation ($\bar{r}$) and the percentage of variance accounted for by sampling error.

## Results and Discussion

Table S31 presents the results of the meta-analysis. Results revealed the mean observed correlation between total metamotivational knowledge and performance was positive and of small to moderate size, $\bar{r}$ = .24, 95% CI [.07, .41]. A similar finding emerged for the relationship between eager knowledge and performance, $\bar{r}$ = .22, 95% CI [.05, .39]. These results are consistent with the main effects found in the main text. The mean observed correlation between vigilant knowledge and performance was small in magnitude and had a confidence interval containing zero, $\bar{r}$ = .10, 95% CI [-.05, .27], suggesting that, at the meta-analytic level, the

relationship may be null. This likely reflects the relatively weaker relation between vigilant knowledge and performance observed in some of the samples.

**Table S31**

Results of Bare-Bones Meta-Analysis

| | $\bar{r}$ | $SD_r$ | $SD_{res}$ | 95% CI | 80% CR |
|---|---|---|---|---|---|
| Total Knowledge | .24 | .11 | .08 | [.07, .41] | [.10, .37] |
| Eager Knowledge | .22 | .11 | .08 | [.05, .39] | [.09, .34] |
| Vigilant Knowledge | .10 | .10 | .07 | [−.05, .27] | [.00, .22] |

*Note:* $k$ = 4 (number of studies contributing to meta-analysis); $N$ = 704 (total sample size); $\bar{r}$ = mean observed correlation; $SD_r$ = observed standard deviation of $r$; $SD_{res}$ = residual standard deviation of $r$; CI = confidence interval around $\bar{r}$; CR = credibility interval around $\bar{r}$.

## References


Ayduk, Ö., & Kross, E. (2010). From a distance: Implications of spontaneous self-distancing for adaptive self-reflection. *Journal of Personality and Social Psychology, 98*(5), 809–829. https://doi.org/10.1037/a0019205

Bateman, T. S., & Crant, J. M. (1993). The proactive component of organizational behavior. *Journal of Organizational Behavior 14*(2), 103-118.

Brown, K. W., & Ryan, R. M. (2003). The benefits of being present: Mindfulness and its role in psychological well-being. *Journal of Personality and Social Psychology, 84*(4), 822–848. https://doi.org/10.1037/0022-3514.84.4.822

Burton, C. L., & Bonanno, G. A. (2016). Measuring ability to enhance and suppress emotional expression: The Flexible Regulation of Emotional Expression (FREE) Scale. *Psychological Assessment*, *28*(8), 929–941. https://doi.org/10.1037/pas0000231

Carver, C. S., & White, T. L. (2013). Behavioral avoidance/inhibition (BIS/BAS) scales. Measurement Instrument Database for the Social Science. Retrieved from www.midss.ie

Corr, P. J. and Cooper, A. J. (2016). The Reinforcement Sensitivity Theory of Personality Questionnaire (RST-PQ): Development and validation. *Psychological Assessment, 28(*11), pp. 1427-1440. https://doi.org/10.1037/pas0000273

Donnellan, M. B., Oswald, F. L., Baird, B. M., & Lucas, R. E. (2006). The Mini-IPIP Scales: Tiny-yet-effective measures of the Big Five Factors of Personality. *Psychological Assessment, 18*(2), 192–203. https://doi.org/10.1037/1040-3590.18.2.192

Duckworth, A. L., & Quinn, P. D. (2009). Development and validation of the Short Grit Scale (GRIT–S). *Journal of Personality Assessment, 91*(2), 166-174. https://doi.org/10.1080/00223890802634290

Dweck, C. S., & Leggett, E. L. (1988). A social-cognitive approach to motivation and personality. *Psychological Review*, *95*(2), 256–273. https://doi.org/10.1037/0033-295X.95.2.256

Elliot, A. J., & Murayama, K. (2008). On the measurement of achievement goals: Critique, illustration, and application. *Journal of Educational Psychology, 100*(3), 613–628. https://doi.org/10.1037/0022-0663.100.3.613

Fishbach, A., Friedman, R. S., & Kruglanski, A. W. (2003). Leading us not into temptation: Momentary allurements elicit overriding goal activation. *Journal of Personality and Social Psychology, 84*(2), 296–309. https://doi.org/10.1037/0022-3514.84.2.296

Freitas, A. L., & Higgins, E. T. (2002). Enjoying goal-directed action: The role of regulatory fit. *Psychological Science, 13,* 1–6. http://dx.doi.org/ 10.1111/1467-9280.00401

Freitas, A. L., Liberman, N., Salovey, P., & Higgins, E. T. (2002). When to begin? Regulatory focus and initiating goal pursuit. *Personality and Social Psychology Bulletin, 28,* 121–130. http://dx.doi.org/10 .1177/0146167202281011

Gross, J. J., & John, O. P. (2003). Individual differences in two emotion regulation processes: Implications for affect, relationships, and well-being. *Journal of Personality and Social Psychology, 85*(2), 348–362. https://doi.org/10.1037/0022-3514.85.2.348

Higgins, E. T., Friedman, R. S., Harlow, R. E., Idson, L. C., Ayduk, O. N., & Taylor, A. (2001). Achievement orientations from subjective histories of success: Promotion pride versus prevention pride. *European Journal of Social Psychology, 31,* 3–23. http://dx.doi.org/10.1002/ejsp.27

Higgins, E. T., Roney, C. J. R., Crowe, E., & Hymes, C. (1994). Ideal versus ought predilections for approach and avoidance: Distinct self-regulatory systems. *Journal of Personality and Social Psychology, 66,* 276–286. http://dx.doi.org/10.1037/ 0022-3514.66.2.276

James, S. A., Hartnett, S. A., & Kalsbeek, W. D. (1983). John Henryism and blood pressure differences among Black men. *Journal of Behavioral Medicine, 6*(3), 259–278. https://doi.org/10.1007/BF01315113

Kashdan, T. B., Disabato, D. J., Goodman, F. R., Doorley, J. D., & McKnight, P. E. (2020). Understanding psychological flexibility: A multimethod exploration of pursuing valued goals despite the presence of distress. *Psychological Assessment, 32*(9), 829–850. https://doi.org/10.1037/pas0000834

King, R. B. (2019). Growth mindsets of motivation and academic engagement. *Unpublished data*.

Lai, K. & Green, S. B. (2016). The problem with having two watches: Assessment of fit when RMSEA and CFI disagree. *Multivariate Behavioral Research, 51*(2-3), 220-239. https://doi.org/10.1080/00273171.2015.1134306

Litman, L., Robinson, J., & Abberbock, T. (2017). TurkPrime.com: A versatile crowdsourcing data acquisition platform for the behavioral sciences. *Behavior Research Methods, 49*(2), 433–442.

Little, T. D., Lindenberger, U., & Nesselroade, J. R. (1999). On selecting indicators for multivariate measurement and modeling with latent variables: When "good" indicators are bad and "bad" indicators are good. *Psychological Methods, 4*(2), 192–211. https://doi.org/10.1037/1082-989X.4.2.192

MacCann, C., Duckworth, A. L., & Roberts, R. D. (2009). Empirical identification of the major facets of conscientiousness. *Learning and Individual Differences, 19*(4), 451–458. https://doi.org/10.1016/j.lindif.2009.03.007

Moss, A., & Litman, L. (2020, August 10). *After the Bot Scare: Understanding What's Been Happening With Data Collection on MTurk and How to Stop It.* CloudResearch. https://www.cloudresearch.com/resources/blog/after-the-bot-scare-understanding-whats-been-happening-with-data-collection-on-mturk-and-how-to-stop-it/.

Scholer, A. A., & Miele, D. B. (2016). The role of metamotivation in creating task-motivation fit. *Motivation Science*, *2*(3), 171–197. https://doi.org/10.1037/mot0000043

Seibert, S. E., Crant, J. M., & Kraimer, M. L. (1999). Proactive personality and career success. *The Journal of Applied Psychology*, *84*(3), 416–427. https://doi.org/10.1037/0021-9010.84.3.416

Snyder, C. R., Harris, C., Anderson, J. R., Holleran, S. A., Irving, L. M., Sigmon, S. T., Yoshinobu, L., Gibb, J., Langelle, C., & Harney, P. (1991). The will and the ways: Development and validation of an individual-differences measure of hope. *Journal of Personality and Social Psychology, 60*(4), 570–585. https://doi.org/10.1037/0022-3514.60.4.570

Tangney, J. P., Baumeister, R. F., & Boone, A. L. (2004). High self-control predicts good adjustment, less pathology, better grades, and interpersonal success. *Journal of Personality, 72*, 271–324. http://dx.doi.org/10 .1111/j.0022-3506.2004.00263.x
